# A Perspective on the Application of Covalent Organic Frameworks for Detection and Water Treatment

**DOI:** 10.3390/nano11071651

**Published:** 2021-06-23

**Authors:** Cristina Arqueros, Félix Zamora, Carmen Montoro

**Affiliations:** 1 Departamento de Química Inorgánica, Universidad Autónoma de Madrid, 28049 Madrid, Spain; cristina.arqueros@uam.es; 2 Institute for Advanced Research in Chemical Sciences (IAdChem), Universidad Autónoma de Madrid, 28049 Madrid, Spain; 3 Condensed Matter Physics Center (IFIMAC), Universidad Autónoma de Madrid, 28049 Madrid, Spain

**Keywords:** covalent organic frameworks, water treatment, detection, toxic metals, membranes, desalination

## Abstract

Global population growth and water resource scarcity are significant social problems currently being studied by many researchers focusing on finding new materials for water treatment. The aim is to obtain quality water suitable for drinking and industrial consumption. In this sense, an emergent class of crystalline porous materials known as Covalent-Organic Frameworks (COFs) offers a wide range of possibilities since their structures can be designed on demand for specific applications. Indeed, in the last decade, many efforts have been made for their use in water treatment. This perspective article aims to overview the state-of-the-art COFs collecting the most recent results in the field for water detection of pollutants and water treatment. After the introduction, where we overview the classical design strategies on COF design and synthesis for obtaining chemically stable COFs, we summarize the different experimental methodologies used for COFs processing in the form of supported and free-standing membranes and colloids. Finally, we describe the use of COFs in processes involving the detection of pollutants in water and wastewater treatment, such as the capture of organic compounds, heavy metals, and dyes, the degradation of organic pollutants, as well as in desalination processes. Finally, we provide a perspective on the field and the potential technological use of these novel materials.

## 1. Introduction

Water is a vital resource to sustain our planet; indeed, about 71% of the Earth’s surface is covered with water. However, it is estimated that 99% of terrestrial water, located in seas, oceans, glaciers, and the atmosphere, cannot be used. Of the remaining 1%, surface water is considered to constitute 0.0067% of total water, with rivers being the most widely used water source. The industry consumes 40% for production processes, cooling, heating, etc. This fact, together with its increasing scarcity, makes the availability of quality water a problem worldwide. Therefore, obtaining drinking water from water resources management in general and industrial/wastewater treatment is a challenge posed to solve this problem.

As we find it in nature, water cannot be used directly for human consumption or industry since it is not pure enough. The presence of pollutants, e.g., ions and substances dissolved as it passes through the soil or air, requires water treatment before use. These treatments involve the combined application of different technologies. Membrane technology has undergone significant development since it offers technically more straightforward and energetically more favorable solutions than conventional separation processes. Polymers are the most common materials used for this purpose. They provide high processability and wide versatility. The available polymers can be functionalized to give rise to novel structures. However, they show low selectivity and permeability because of their low chemical and thermal stability and their amorphous nature. These problems could be solved using crystalline and tunable structures.

**Figure 1:**
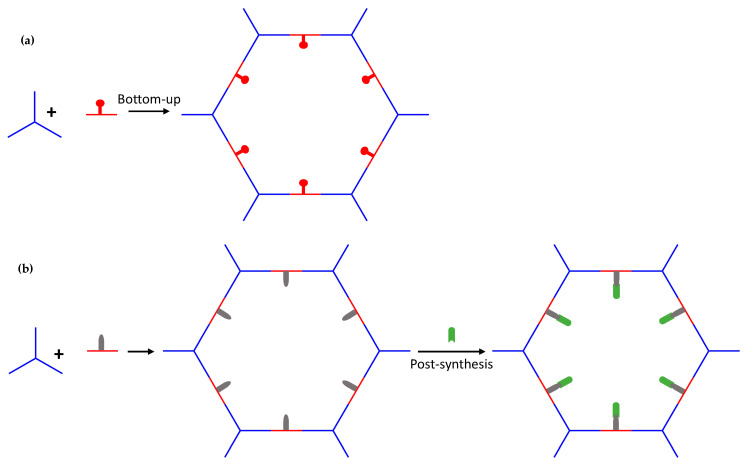
Examples of reticular design strategies: (**a**) variation of the pore sizes of the structures and (**b**) introducing functional groups, either pre- or post-synthetically.

In this sense, Covalent Organic Frameworks (COFs) are a new emerging class of crystalline materials with high permanent porosity, low density, and high thermal and/or chemical stability. COFs are based on organic structures formed by the linkage of molecules using dynamic covalent bonds [1]. Nowadays, research in this field is exponentially growing, collecting already more than 700 COFs [2]. Its modular nature allows controlling the topology and features of their pores by rationally choosing the molecular organic precursors or introducing functional groups, either pre- or post-synthetically, for specific applications. Then, both the variation of the pore sizes of the structures and the introduction of functional groups, pre- or post-synthetically, allow the COFs design to remove the ions present in contaminated waters that cause fouling and corrosion (Figure 1) [3].

In short, the design of *à la carte* COFs offers an added value, since it opens the possibility of developing customized treatments based on the initial and final quality required. This allows optimizing the operation, performance, and consumption of resources, something not currently available in the target market.

**Figure 2:**
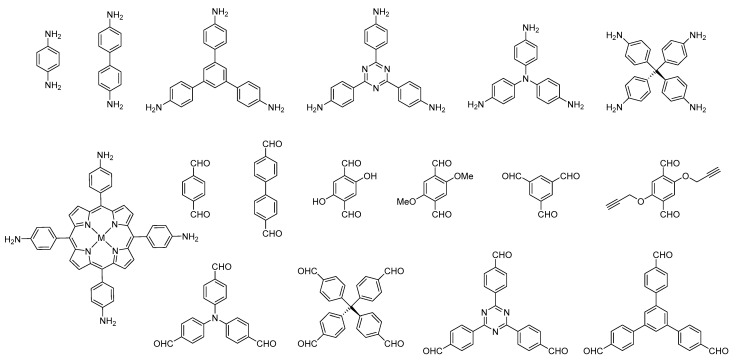
Selected examples of the most common initial molecular precursors used for the preparation of 2D and 3D imine-based COFs.

The typical formation of COFs takes place using condensation reactions [4]. Thus, the first COFs were obtained from the co-condensation reaction of boronic acids and catechol and the self-condensation of boronic esters. Later, the Schiff-based reaction between primary amines and aldehydes gave rise to the formation of imine, hydrazone, and azine-based COFs. The construction of a 2D or 3D COF networks is based on the molecular precursor geometries that will define the building of a stacked or interconnected structure. The most common initial molecular precursors used to prepare 2D and 3D COFs are shown in Figure 2. Excellent reviews have collected additional information on COFs design [1].

For water treatment applications, the chemical stability of the COFs is a crucial issue to be considered [5]. In this regard, the first COFs obtained based on boroxine, and boronate ester formation showed the disadvantage of being unstable against moisture [6,7]. It was due to the presence of boron sites along with their structures. These are sensitive sites to nucleophilic attack, which limits the applications of this kind of COF. It was not until 2011 that Wang et al. [8] reported the first imine-based COF, which showed exceptional stability in water and most organic solvents due to the structural strength offered by the electron-rich imine linkage. This fact encouraged research towards the search for new stable COFs towards hydrolysis such as hydrazine-linked, triazine-linked, azine-linked, and ketoenamine-linked COFs [9,10]. It is highlighting that ketoenamine COFs are also highly stables in both acid and basic solvents. Then, the number of possible applications for these materials increased considerably. Synergic strategies have also been developed to increase chemical stability, as the introduction of specific functional groups such as methoxy or hydroxy groups in the organic linker [11]. An excellent review article has been recently published showing COF evolution [12]. Another problem that must be faced when establishing a possible application for COFs is their limited processability. Usually, COFs are obtained in the form of insoluble powders. Shaping of COFs is a very important issue to be considered for the final application [13,14]. In this sense, recently, our research group has developed a novel strategy for the preparation of COF aerogel monoliths that were used as a proof-of-concept in the removal of organic contaminants from water [15,16].

On the other hand, in the last decade, the number of reports on the use of COFs for the preparation of free-standing membranes, supported on porous surfaces (thin-film composite, TFC, membrane) or integrated into polymeric matrices to form hybrid membranes (mixed matrix membranes, MMMs), has increased considerably. Thus, membranes based on COFs have been suggested for applications that mainly involve the separation of gases (e.g., mixtures containing CO_2_ [17,18], H_2_ [19], hydrocarbons [20], air purification), liquids (wastewater treatment, reverse osmosis, pervaporation, nanofiltration) or as proton exchange membranes in fuel cells [21,22].

Although some authors have recently reviewed the use of COFs for water treatment [23,24,25], in this perspective article, apart from collecting the state-of-the-art membranes based on COFs for this purpose from the last five years, we propose possible future strategies to carry out. Moreover, considering the importance of the mechanical stability of COF-based membranes, we have classified the preparation methods according to whether they are obtained as free-standing or supported membranes. Finally, for the first time, it is also collected in the same manuscript the potential application of COFs in the detection of pollutants in water such as organic compounds, heavy metals, and dyes, as well as in the degradation of organic pollutants.

## 2. COF Processing

As mentioned above, COFs processability is a significant challenge that must be solved before their use in water treatment. This section shows different strategies reported to date that allow the implantation of COFs for this purpose, either in the form of membranes or processed in some other way such as suspensions, monoliths, or pellets.

### 2.1. COF-Based Membranes

**Figure 3:**
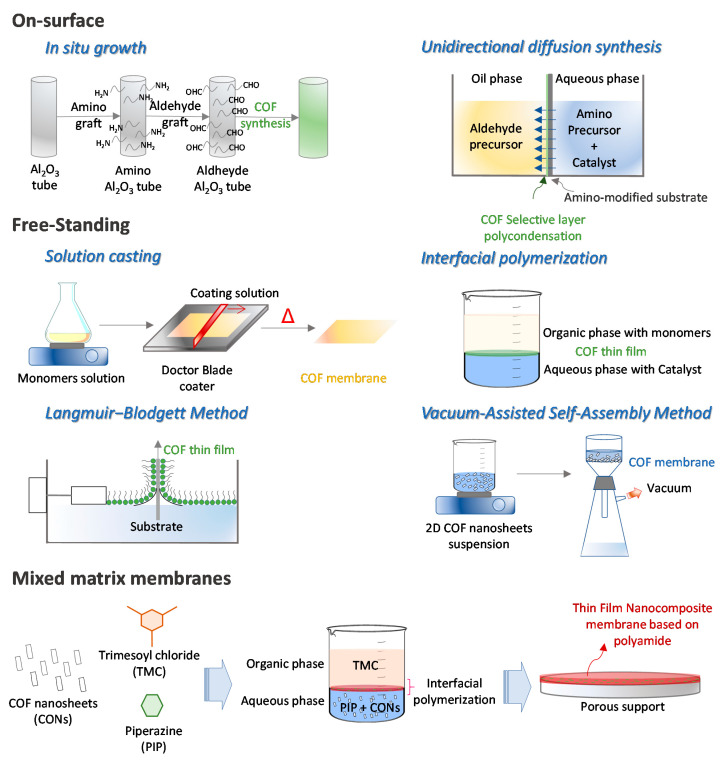
Schematic representation of the COF-based membrane preparation strategies.

With the aim of replacing the current polymeric membranes used for water treatment, different approaches have been reported in the preparation of COF-based membranes. These approaches differ depending on whether the COF-based membrane has been prepared free-standing, with the help of some porous support (on-surface), or as hybrid membranes in which COF acts as filler of a polymer matrix, giving rise to what is known as mixed matrix membrane (MMM) (Figure 3).

#### 2.1.1. On Surface

- In situ growth

The in situ growth is one of the first methods used for the preparation of imine-based COF membranes. It consists of the obtention of a continuous COF layer, usually under solvothermal conditions, that grows on a previously functional surface. 

**Figure 4:**
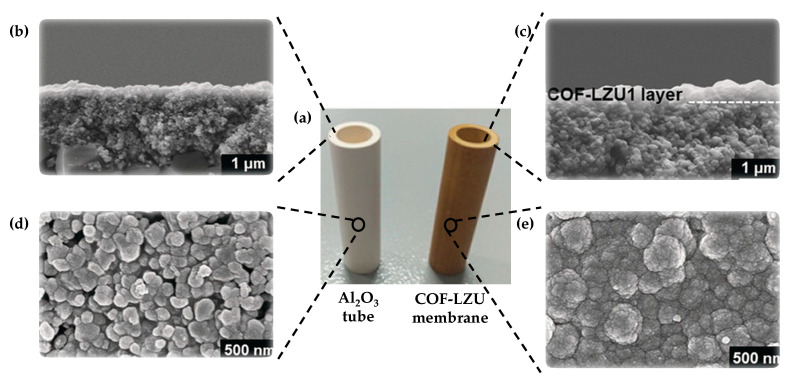
(**a**) Photographs of an untreated Al_2_O_3_ tube and a tubular COF-LZU1 membrane. (**b**,**c**) Cross-sectional SEM images of an untreated Al_2_O_3_ tube (**b**) and a tubular COF-LZU1 membrane (**c**). (**d**,**e**) Top-view SEM images of an untreated Al_2_O_3_ tube (**d**) and a tubular COF-LZU1 membrane (**e**). Reprinted with permission from [26]. Copyright (2018) John Wiley and Sons publications.

Following this strategy, Fan et al. [26] were the pioneers in obtaining a robust 2D imine-linked COF-LZU1 membrane (400 nm of thickness) on the outer modified surface of alumina tubes (Figure 4). Particularly, these authors pre-grafted with 3-aminopropyltriethoxysilane (APTES) and aldehyde groups the alumina surface to create an anchor between the ceramic support and the COF. Then, they placed the amino-functionalized alumina tube surface into a Teflon tube, which also contained dimethylformamide (DMF) and the reagents 1,3,5-triformylbenzene (TFB) and p-phenylenediamine (PDA). After the solvothermal condensation reaction took place, a continuous and stable COF-LZU membrane was formed on the outer surface of the alumina tube. This membrane showed excellent water permeability and reasonable rejection rates (above 90%) for dye molecules. Alternatively, other groups have prepared COF-5 [27] and COF-300 [28] membranes with 1 μm and 5.5 μm on modified porous ceramic support by microwave irradiation and solvothermal conditions, respectively.

The use of polymeric substrates has also been tested in the preparation of COF-based membranes. This kind of support presents the advantage of being cheaper and easier to handle. However, they require mild working conditions. In this sense, Jiang et al. [29] were able to grow the imine-based COF TpPa-1 on polyacrylonitrile (PAN) under environmental conditions. For that, after the support was functionalized with aldehyde groups, it was immersed into the mixture of 1,3,5-triformylphloroglucinol (Tp) and p-phenylenediamine (Pa-1) in water and acetic acid. The imine condensation reaction was carried out at room temperature, and the so-formed TpPa-1 membrane (thickness ~65 nm) was formed and used for dyes separation.

In some cases, after the in situ growth, a secondary growth method is applied to obtain a continuous and well-intergrown membrane. Thus, Huang et al. [30] got to prepare IISERP-COF1 membranes on an APTES-modified αAl_2_O_3_ tube by the epitaxial growth of the previously deposited seeds in the form of tiny crystals.

- Unidirectional diffusion synthesis

This strategy was designed by Wang et al. [31] for the preparation of COF membranes directly onto a porous support. This procedure consists of using a diffusion cell divided into two parts by a porous support. One of the COF precursors is located at one side of the diffusion cell, while a solution containing the other COF precursor and the catalyst is on the other side. Then, the unidirectional diffusion flow occurs due to the concentration gradient or the permeability of the substrate, causing the formation of COFs selective layers on it. Following this strategy, they fabricated a TpPa membrane onto a (polyvinylidene fluoride) PVDF porous support. For that, p-phenylenediamine (Pa) aqueous solution with the catalyst was deposited in one side of the diffusion cell while 1,3,5-triformylphloroglucinol (Tp) n-hexane solution was deposited in the other. The diffusion occurs since Pa molecules can pass through the PVDF support and react with Tp molecules to give rise to the TpPa/PVDF membrane. This procedure allows the preparation of membranes with high performances for dye rejections thanks to the excellent adhesion between COF layers and support. Later on, to obtain COF thin-films for desalination, these authors applied a secondary growth to the unidirectional diffusion synthesis [32].

#### 2.1.2. Free-Standing

- Solution Casting

**Figure 5:**
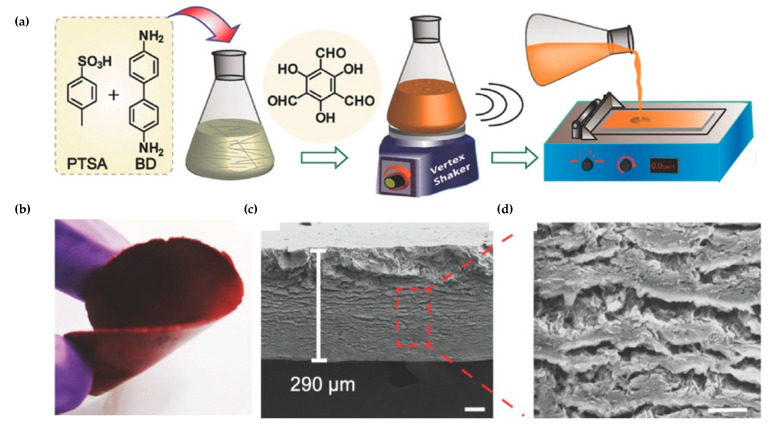
(**a**) Schematic representation of the M-TpBD membrane fabrication. (**b**) Photographs of M-TpBD membrane demonstrating its flexibility. SEM images showing (**c**) the cross-section M-TpBD and (**d**) the corresponding zoomed view 10 μm. Reprinted with permission from [33]. Copyright (2016) John Wiley and Sons publications.

Solution casting was used by Banerjee et al. [33] for the preparation of defect- and crack-free ketoenamine COF membranes (Figure 5) (thickness 200–700 µm) that were tested for selective molecular sieving. In this case, reagents were well-mixed and scattered onto a clean glass plate before their baking. Then, the membrane was peeled off using distilled water and acetone to remove the unreacted reagents.

This strategy has many advantages since it is a simple and effective method that allows obtaining free-standing membranes on a large scale, with adjustable thickness, and at low cost. However, its use is still limited to the formation of some β-ketoenamine COF membranes.

- Interfacial Polymerization

Interfacial polymerization (IP) allows the obtention of free-standing thin-films at the liquid-liquid, solid-liquid, or air-liquid interface, which can act as active layers when they are transferred on porous support to give rise to a thin-film composite (TFC) nanofiltration membrane. This technique has been widely used in the preparation of polyamides and polyesters thin-films since it is scalable and able to produce selective layers (50–250 nm) highly permeable to water [34,35]. In the case of the preparation of free-standing thin-film COFs, most monomers are insoluble in water, limiting the use of molecules.

**Figure 6:**
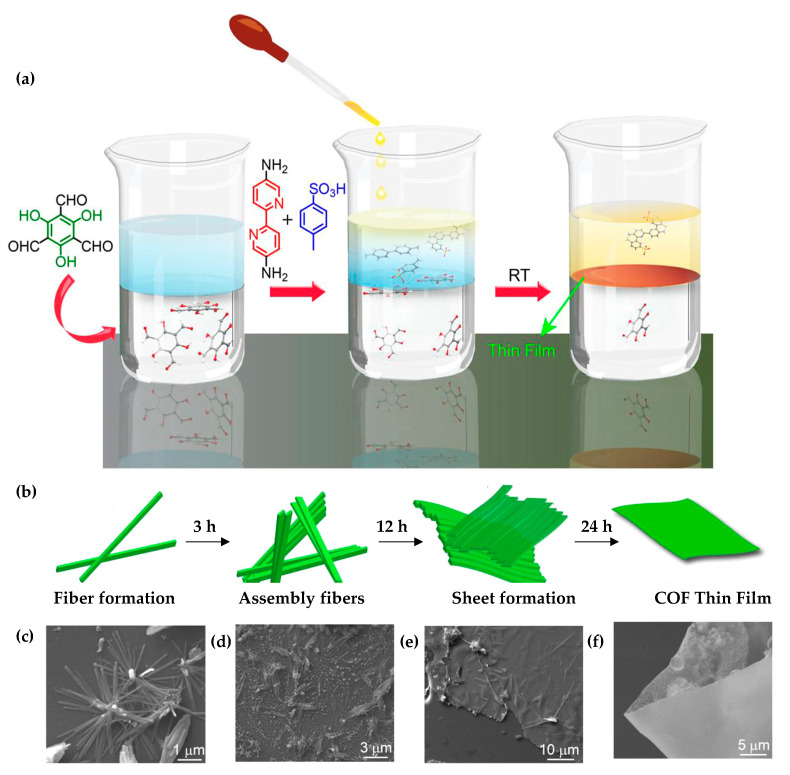
(**a**) Schematic representation of the interfacial crystallization process used to synthesize the Tp-Bpy thin-film. (**b**) Proposed mechanism for the COF thin-film formation (Tp-Bpy). (**c**–**f**) SEM images of materials obtained at different stages (fiber formation, assembled fibers, and sheet formation) of thin-film formation, respectively. Reprinted with permission from [36]. Copyright (2017) American Chemical Society.

Banerjee et al. [36] were pioneers in the preparation of free-standing imine-based COF films with different pore sizes at the water-organic interface at room temperature. The crystallization problem was solved by controlling the diffusion rate at the interface. They used amine-p-toluene sulfonic acid (PTSA) salt as the catalyst and a substitute for free amine in the aqueous phase, while the organic phase contained the aldehyde (Figure 6a). Furthermore, these authors studied the formation mechanism of the thin-films by SEM images (Figure 6b,c), showing that the formation of the thin-film COF occurs after the assembly of fibers and sheets.

A similar approach was reported by Dichtel et al. [37], but in this case, both monomers (1,3,5-tris(4-aminophenyl)benzene and terephthalaldehyde) were dissolved in the organic phase while the water contained the Lewis-acid catalyst, Sc(OTf)_3_. TAPB-PDA COF free-standing membrane with a tunable thickness (2.5 nm to 100 mm) was obtained at the interface of both solvents and transferred onto poly(ether sulfone) (PES) supports to be tested in dye removal from wastewater. Mariñas et al. [38] grew multi-active layers of COF (1, 3, and 6 layers thickness ~10 nm per layer) through IP of terephthalaldehyde and tris(4-aminophenyl)benzene monomers near the surface of a PES ultrafiltration membrane support. PES was previously impregnated with an aqueous solution of the catalyst, and a solution of the monomers on a mixture of organic solvents was deposited on the top. After the polyamine COF thin-film polymerization took place at the aqueous/organic interface, the solvents were removed by suction, and the COF thin-film was deposited directly on the support.

**Figure 7:**
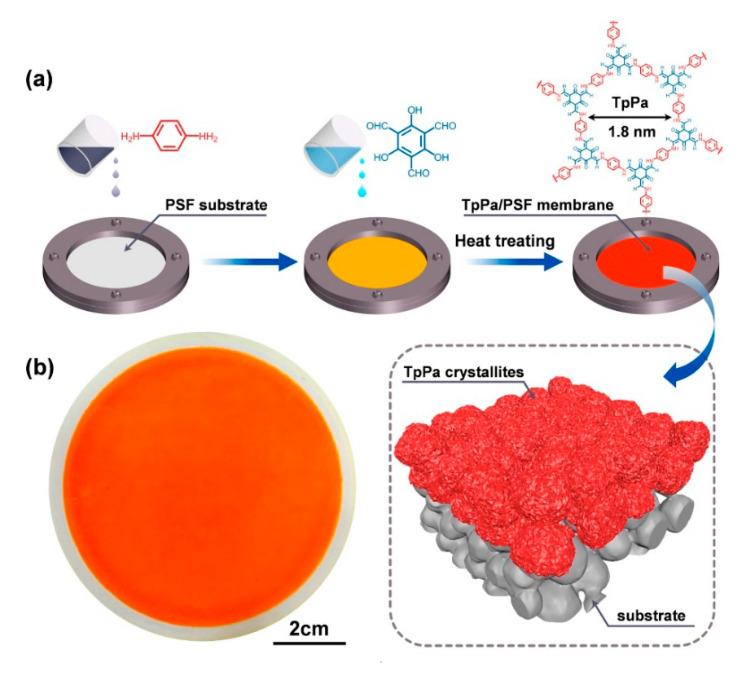
Fabrication process of the TpPa/PSF membranes via IP. (**a**) Schematic illustration for the preparation of the TpPa/PSF composite membranes. (**b**) Physical appearance of the as-synthesized TpPa/PSF composite membrane. Reprinted from [39]. Copyright (2018), with permission from Elsevier.

With the aim to avoid the difficulty in the transference of the thin-film layer and the adhesion problems, Wang et al. [39] established a new methodology based on IP that allows the formation of the active layer directly on the porous support. This strategy is carried out under mild conditions and consists of the impregnation of polysulfone support, first with a solution of 1,3,5-triformylphloroglucinol (Tp) in n-hexane and later with a solution of p-phenylenediamine (Pa) and acetic acid in water. After 1 minute of reaction, they obtained highly permselective TpPa/PSF composite membranes (Figure 7). This method was used recently by Lang et al. [40] for the preparation of COF-LZU1 on PES microfiltration membranes.

- Langmuir−Blodgett (LB) Method

This method allows the obtention of thin-film membranes with large dimensions and tunable thicknesses that can be easily transferred onto different substrates; however, it is still a little developed procedure for COF membrane fabrication. For the first time, Lai et al. [41] used it to prepare a crystalline 2D COF membrane of 4 unit cells of thickness at the water−air interface. In this case, the β-ketoenamine linkage occurs between 1,3,5-triformylphloroglucinol (TFP) and 9,9-dihexylfluorene-2,7-diamine (DHF) on the surface of the water. The reaction was initiated by using trifluoroacetic acid and through the compression of the surface layer. The resulting TFP-DHF-COF thin-film was deposited onto an anodic aluminum oxide (AAO) porous support and applied for organic solvent nanofiltration thanks to its notable permeability.

- Vacuum-Assisted Self-Assembly Method

**Figure 8:**
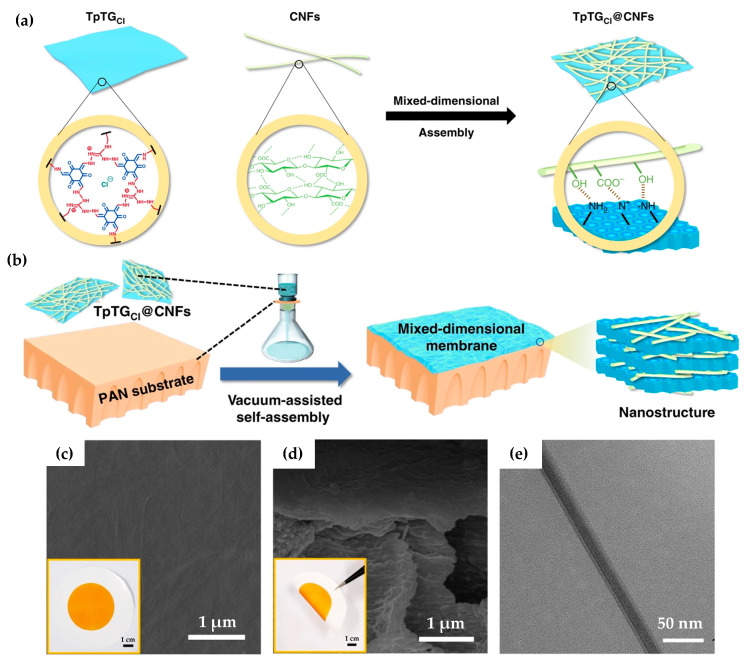
(**a**) Scheme of the assembly of TpTGCl and CNFs. (**b**) Scheme of the vacuum-assisted self-assembly method. (**c**) Surface SEM image of the TpTGCl@CNFs-5/PAN membrane inserted with a digital photo of the membrane. (**d**) Cross-sectional SEM image of the TpTGCl@CNFs-5/PAN membrane inserted a tweezer bent membrane photo. (**e**) Cross-sectional TEM image of the TpTGCl@CNFs-5 membrane [42].

Following the reported methodology for preparing graphene and other related 2D materials membranes, some researchers have obtained membranes based on COF nanosheets. In this case, previous strategies such as chemical or mechanical exfoliation have to be done to obtain 2D COF nanosheets from the bulk COF material. Then, the vacuum filtration of these nanosheets allows obtaining a COF membrane. However, the mechanical stability of the membranes, due to the weak π–π interactions between the COF nanosheets, has to be enhanced. In this sense, Jian et al. [42] have prepared a series of COF membranes from the assembly of 2D COF nanosheets and 1D cellulose nanofibers (CNFs) (Figure 8). It was possible because of the intrinsic positive charge of guanidinium units of the COF TpTG_Cl_, synthesized by the reaction between triformylphloroglucinol (Tp) and triaminoguanidinium chloride (TG_Cl_), and the negatively charged carboxyl groups on CNFs. Later on, the COF membrane was prepared by the vacuum-assisted self-assembly method on PAN support. The good compaction of both structures allowed us to obtain a uniform, flexible, and stable membrane with a pore size ranged between 0.45–1 nm, which found a potential application in molecular separation such as alcohol dehydration, dye, and salt rejection.

#### 2.1.3. Mixed Matrix Membranes

Another strategy for obtaining COF-based membranes consists of dispersing these materials within a polymeric matrix to give rise to what is known as mixed matrix membranes or hybrid membranes. The use of COFs as filler compared with classical inorganic materials such as zeolites, graphite, metal-organic frameworks (MOFs), and carbon nanotubes helps to avoid particle agglomerations and the consequent deposition since they improve the compatibility with the polymer due to its purely organic nature. Moreover, the polymer matrix confers mechanical stability and flexibility to the resulting membrane.

**Figure 9:**
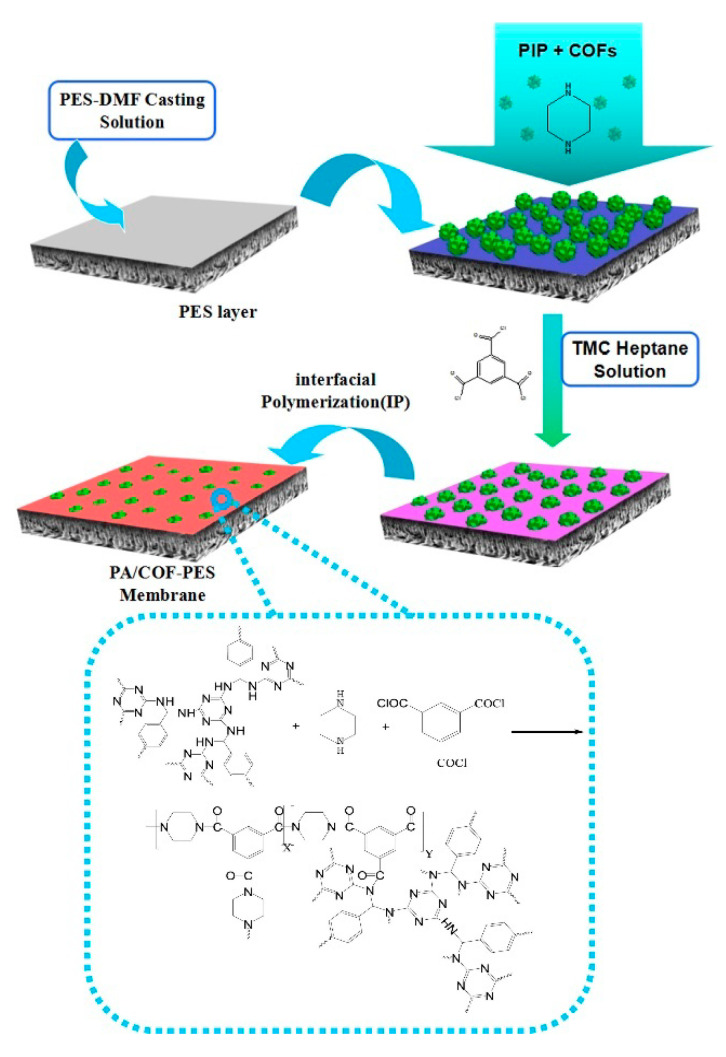
Fabrication process of the TFN membrane. Reprinted from [43]. Copyright (2017), with permission from Elsevier.

One of the first works that used COFs as fillers in the preparation of thin-film nanocomposite (TFN) membranes for nanofiltration was reported by Wu et al. [43]. Specifically, these authors carried out an interfacial polymerization process between the COF SNW-1, characterized by secondary amine groups, monomers trimesoyl chloride (TMC), and piperazine (PIP). In the first step, SNW-1 was dispersed in an aqueous solution of PIP and then filtrated on the surface of polyether sulfone (PES) support layer. Later, the polyamide layer was formed after submerging it in the organic phase composed of TMC in n-hexane (Figure 9). 

Later on, in a similar study, these authors reported the preparation of TFN membranes through COF nanosheets, which showed good performance in water desalination [44]. Once again, the presence of amino and hydroxyl groups in the COF nanosheets favored formatting hydrogen bonds between them and PIP in the aqueous solution, which decreased the diffusion rates and consequently improved the quality of the membrane as well as its hydrophilicity. The use of COF nanosheets as filler for the formation of TFN membranes has also been reported by other groups [45,46].

Recently, Xia et al. [47] have reported a novel approach for growing a COF inside the pores of the polymer matrix through interfacial polymerization. In a first step, the porous PVDF ultrafiltration membrane was filled with acetic acid, which acts as a catalyst of the imine bond formation reaction. Then, a mixture of tiformylbenzene (TFB) and p-phenylenediamine (PDA) in 1,4-dioxane was poured onto the membrane surface, giving rise to the COF formation after a certain period. This strategy ensures a homogeneous distribution of the COF in the polymer matrix. Besides, the hydrophilic properties of COF give the membrane higher permeability and an improvement in selectivity towards dyes.

### 2.2. Nanoprocessed COFs

**Figure 10:**
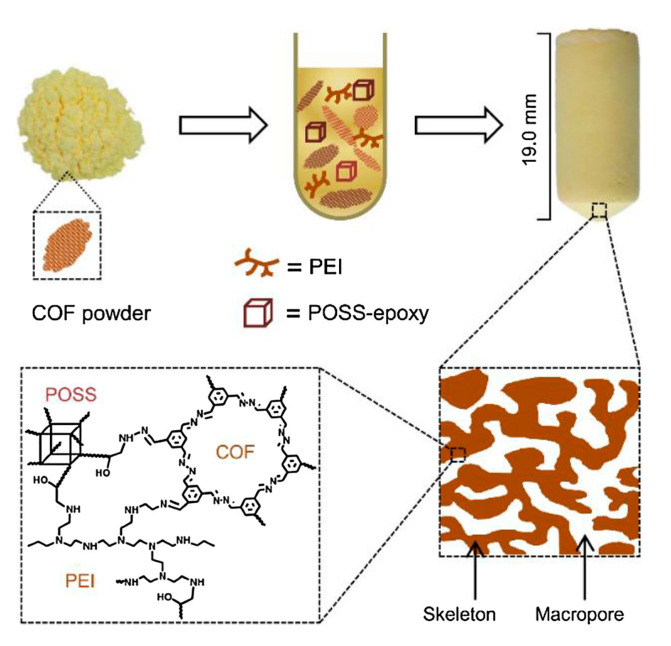
Construction of the hierarchically porous monoliths from the COF powder. Reprinted from [48]. Copyright (2018), with permission from Elsevier.

Apart from the preparation of COF-based membranes, some COFs are processed into pellets, colloids, or monoliths before their final application. For example, in 2018, Z. Liu et al. [48] reported constructing hierarchically porous monoliths of COFs for bisphenol A and other hydrophilic pollutants’ absorption. This research group developed a method based on shaping COF powder, synthesized by reacting 1,3,5-triformylbenzene and hydrazine hydrate, into monoliths. This approach preserves the crystallinity of the COF in the monolith and creates new macropores with sizes ranging from 0.43 to 3.51 μm. The method is based on a ring-opening polymerization of amine groups with epoxy groups by introducing azine-linked COF into fresh pre-polymerization solution (FPS) (Figure 10). FPS consists of a mix of polymers, including poly(ethylenimine) (PEI), octaglycidyldimethylsilyl POSS (POSS-epoxy), poly(ethylene glycol) (PEG), and others.

Very recently, our research group has reported the synthesis of a series of monolithic aerogel imine-based COFs following a three-step method [15]. For that, in the first step, it is prepared a COF gel under mild conditions by a sol-gel process. Then, the COF gel undergoes solvent exchange, and, finally, it is activated with supercritical CO_2_. The resulting materials show very low densities (ca. 0.02 g cm^−3^), high porosities as well as interesting mechanical properties. Specifically, they have an elastic behavior at low pressures (<25–35%) that becomes plastic after applying high pressures. 

## 3. COFs for Water Treatment

### 3.1. COF-Based Membranes for Waste Water Treatment

One of the primary sources of water pollution comes from the textile and dye industries. Most dyes are mutagenic and carcinogenic, causing severe problems for human health and the environment. Then, the preparation of ion/molecule sieving membranes is a challenge that can be solved with the design of COF-based membranes with defined pores.

Using IP strategy, Ma et al. [49] prepared two COF-based membranes with a tunable pore size (from >1 nm to ~0.6 nm) by modifying the 2D COF layer stacking from AA (FS-COM-2) to AB (FS-COM-1). These membranes were tested for filtration experiments of rhodamine B (RhB) molecule and 14 cations. As expected, FS-COM-1 avoided the permeation of all cations and RhB molecule while they can go through FS-COM-2.

**Figure 11:**
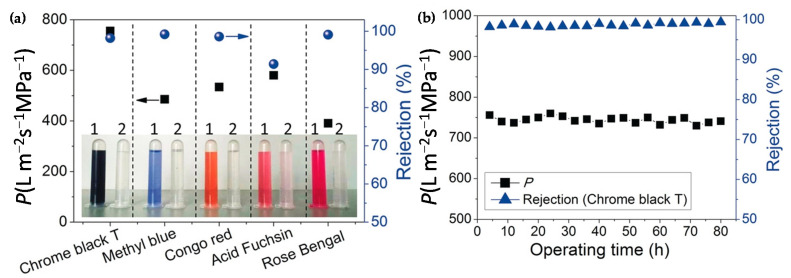
(**a**) Water permeance and rejection rates of the tubular COF-LZU1 membrane in the NF of different dyes (photographs show the colors of the dye solutions before (1) and after (2) NF). (**b**) Stability test of the tubular COF-LZU1 membrane in a long-time NF of CB. Operating pressure: 0.5 MPa; dye concentration: 100 mg L^−1^; room temperature. Reprinted with permission from [26]. Copyright (2018) John Wiley and Sons publications.

COF-LZU1 membrane prepared via in situ growth by Caro et al. [26] showed a water flux permeance of 760 L m^−2^ h^−1^ MPa^−1^. This exceptional value is more than double of the usually reported membranes (~300 L m^−2^ h^−1^ MPa^−1^). The COF-LZU1 membrane was tested in the rejection of five different dyes (chrome black T (CB), methyl blue (MB), acid fuchsin (AF), Congo red (CR), and rose Bengal (RB)). As shown in Figure 11a, even though the pore size of the LZU1 is ca. 1.8 nm, this membrane exhibited high rejections for dye molecules larger than 1.2 nm. The rejection rate for AF was low because of its spherical shape. Particularly, MB and CR revealed rejection rates of 99.2 and 98.6%, respectively. This is possibly due to the aggregation of the dye molecules in the form of larger micelles or the deposition of the COF-LZU1 particles. Moreover, these results are maintained during 80 h of operation time, which shows the high mechanical stability (Figure 11b). So, this membrane can be used to purify dye molecules from saline solutions since it was not selective for hydrated salt ions.

**Figure 12:**
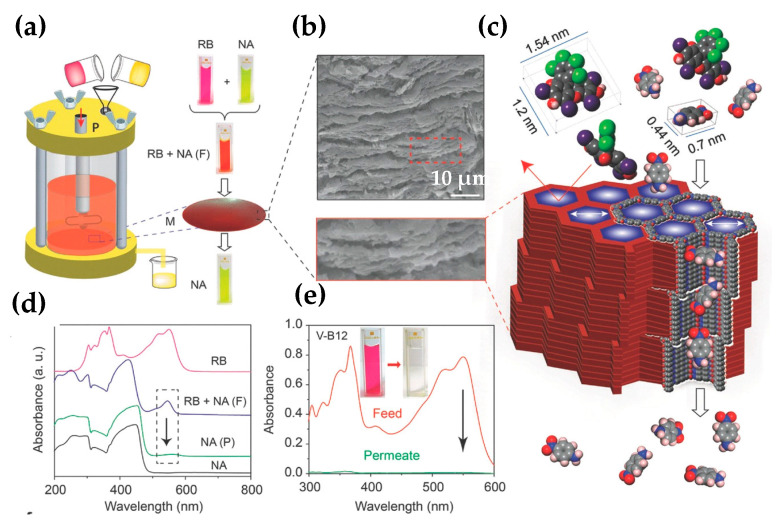
(**a**) Scheme of the nanofiltration assembly and selective molecular separation of NA from a mixture of NA and RB, (“F” denotes feed and “P” for permeate); (**b**) SEM cross-section showing stacked layer sheets in M-TpBD; (**c**) Scheme for molecular sieving mechanism through M-TpBD; (**d**) UV–vis spectra of selective recovery of NA from mixture of RB and NA from water; (**e**) UV–vis spectra of vitamin-B12 (V-B12) and the filtrate water after passing through M-TpBD. Reprinted with permission from [33]. Copyright (2016) John Wiley and Sons publications.

Banerjee et al. [33] also used their M-TpBD and M-TpTD membranes, obtained by solution casting, in the water filtration of the organic dyes, RB, MB, and CR. Especially, M-TpBD showed rejection rates of 99%, 96%, and 94% for RB, CR, and MB, respectively. On the other hand, rejection rates for M-TpTD were lower, mainly 84, 80, and 83% for RB, CR, and MB, respectively, due to its bigger pore size. With the aim to confirm the size exclusion property of M-TpBD, they prepared a solution with RB and nitroaniline (NA) (Figure 12). The filtration of this mixture gave rise to permeating the NA, and the rejection of RB, since the molecular size of NA is lower than 1 nm. These results were confirmed by removing different organic molecules (vitamin B12, curcumin, and tetracycline) from various solvents as well as the Gram-negative *Escherichia coli* bacteria in water. 

### 3.2. COF-Based Membranes for Water Desalination

Desalination techniques are usually based on TFN membranes, which consist of an active layer of polyamide (PA) (~100 nm thickness) deposited onto a porous support. These membranes show good performances in the retention of organic compounds or multivalent ions at low pressures. However, there is a need to break the well-known trade-off between flux and selectivity. In this sense, Jiang et al. [50] prepared, by IP of piperazine (PIP) and trimesoyl chloride (TMC), a PA layer with thickness < 10 nm through the previous deposition of 2D COF nanosheets (CONs) on a PES microfiltration membrane via vacuum-assisted assembly method. The extraordinary hydrophilicity and high porosity of the TpPa-1 CONs allowed a homogeneous distribution on the surface of the PIP monomers via hydrogen bonding and an improvement in the assembly of TMC during the IP. The authors studied different CON loadings and observed that the higher the load, the thinner the PA. Consequently, the optimum value was found for 0.5 mg of CONs the PA thickness was ~7 nm, which gave rise to a water flux of 535.5 L m^−2^ h^−1^ MPa^−1^ and a rejection for Na_2_SO_4_ of 94.3%.

**Figure 13:**
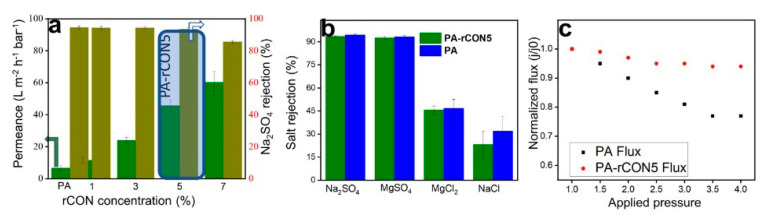
Performance evaluation of membranes: (**a**) desalination of PA and PA−rCON5, (**b**) different salt rejection, and (**c**) flux at different pressure. Reprinted with permission from [44]. Copyright (2020) American Chemical Society.

Recently, the same group [44] reported for the first time a MMM for desalination based on reactive fillers. For that, they made use of reactive CONs (rCONs) of the COF CTF-1, which acted as filler of PA. Thanks to the presence of amine and hydroxyl groups in rCONs linked by hydrogen bonding to PIP, decreasing the diffusion rate and avoiding the formation of the ridges was possible. Furthermore, the interaction between the amino groups and the TMC during the IP enhanced the antifouling properties and hydrophilicity. Then, they studied the performance of this membrane at different rCON loadings and found that the water flux increased with the load (Figure 13a), reaching its maximum value of 46.5 L m^−2^ h^−1^ bar^−1^ for 5 wt %, which was 6.8 times higher than the pristine PA and a rejection factor for Na_2_SO_4_ of 93.5% (Figure 13b). It is highlighted that this flux was maintained at different pressures (Figure 13c). Likewise, they studied the rejection of other salts and found the following rejection order: Na_2_SO_4_ > MgSO_4_ > MgCl_2_ > NaCl which obeys the Donnan theory. This theory postulates that due to the negative charge of the membrane, the divalent anions together with monovalent cations will be repulsed to a greater extent than the divalent anions with divalent cations. In the same way, monovalent anions with mono or divalent cations will show lower rejection rates.

**Figure 14:**
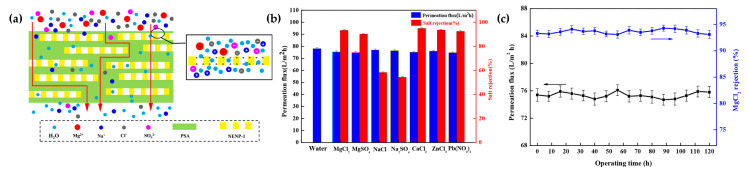
(**a**) Schematic illustration of separation process for NENP-1-PSA/PES nanocomposite membrane; (**b**) Permeation flux and salt rejection of N-3 for separating different salts (1 g L^−1^ aqueous solution, 25 °C); (**c**) Permeation flux and MgCl_2_ rejection of N-3 membrane with different operation time. Reprinted with permission from [46]. Copyright (2020) American Chemical Society.

Alternative solutions have been reported by Li Wang et al. [46], who developed a TFN positively charged membrane that exhibited acid stability and high performances. In this case, they used NENP-1, a triazine structure, as filler of polysulfonamide (PSA), which was synthesized via IP of polyallylamine (PAH) and 1,3-benzenedisulfonyl dichloride (BDSC). The presence of 0.1 w/v% of NENP-1 showed an increase in water flow and MgCl_2_ rejection thanks to the COF hydrophilicity and porosity. Higher concentrations of COFs gave rise to worse values due to particle aggregation. Likewise, the rejection of different types of ions was studied, and the following rejection order was observed at the same permeation flux: CaCl_2_ (94.9%) > ZnCl_2_ (93.8%) > MgCl_2_ (93.3%) > Pb(NO_3_) (92.4%) > MgSO_4_ (90.2%) > NaCl (58.2%) > Na_2_SO_4_ (54.3%) (Figure 14). Again, these results agree with the Donnan effect. The good compatibility between the COF and the polymer matrix was shown by the 120 h of operation time reached.

### 3.3. COF Monoliths for Decontamination of Organic Pollutants

**Figure 15:**
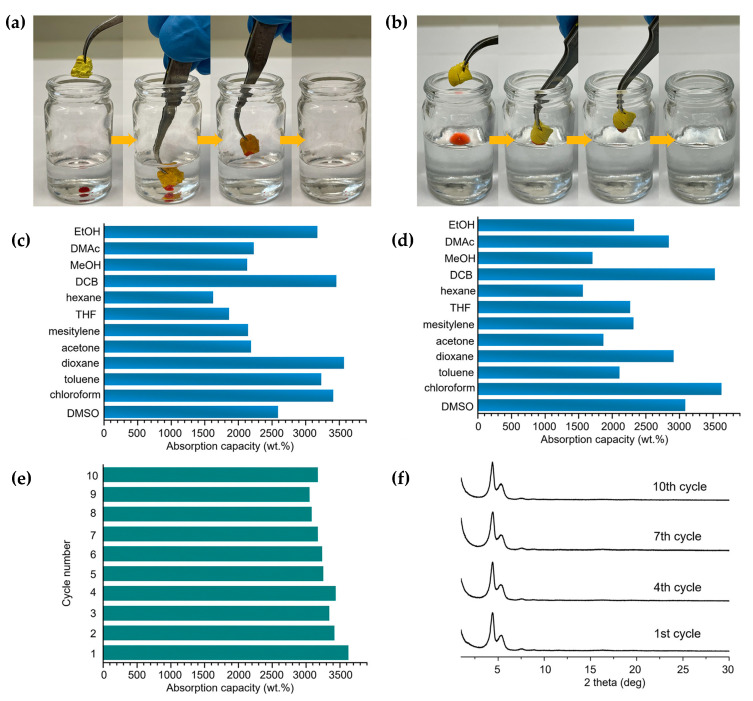
Summary of the performance of TAPB-TFPA and TAPB-OMePDA COF aerogels for the absorption of organic solvents. (**a**,**b**) Digital photographs of absorption of (**a**) dyed chloroform and (**b**) dyed silicon oil from water. (**c**,**d**) Organic solvent absorption capacities of (**c**) TAPB-OMePDA COF aerogel and (**d**) TAPB-TFPA COF aerogel. (EtOH: ethanol; DMAc: dimethylacetamide; MeOH: methanol; DCB: dichlorobenzene; THF: tetrahydrofuran; DMSO: dimethyl sulfoxide). (**e**) Performance of the TAPB-TFPA COF aerogel over multiple cycles of absorption of chloroform and (**f**) PXRD spectra of the recycled TAPB-TFPA COF after multiple cycles of absorption, washing, and drying. Reprinted with permission from [16]. Copyright (2021) American Chemical Society.

Very recent studies have made use of COF aerogels for the decontamination of organic pollutants present in water due to their high surface areas, porosities, as well as low densities. In this sense, our research group reported the use of 3 monolithic COF aerogels as a proof-of-concept for the removal of toluene from contaminated water, showing an efficiency ca. 99% [15]. The results derived from this study showed adsorption kinetics of less than 1 min and a material regeneration capacity of at least 10 cycles. In a similar report, Verduzco et al. [16] described the preparation of six different imine-based COF aerogels. Then, they selected two of them (TAPB-OMePDA and TAPB-TFPA) to be tested as models of absorbents of organic solvents (Figure 15). For that, they placed the COF aerogel on the surface of water containing the corresponding organic phase and observed different absorption capacities depending on the organic solvent used (Figure 15a,d). The best absorption capacities were obtained for dichlorobenzene and chloroform in the case of TAPB-TFPA COF aerogel and dioxane and dichlorobenzene in the case of TAPB-OMePDA COF aerogel, which was maintained after 10 cycles of absorption, washing, and drying (Figure 15e,f). Furthermore, TAPB-TFPA COF aerogel was used in the removal of organic and inorganic micropollutants from water such as Methylene Blue (MB), Methyl Blue (MLB), Congo Red (CR), Methyl Orange (MO), and gold nanoparticles (Au NPs). The best removal efficiencies were obtained in the case of MB (99.0%), followed by MLB (97.2%).

### 3.4. COFs for Degradation

In the last decades, the chemical industry has developed considerably, and a variety of new chemical pollutants have entered our daily lives, but most importantly, in the environment. Recently, the use of advanced oxidation processes (AOPs) has been reported for the degradation of dyes or Bisphenol A, which are common organic pollutants in wastewater nowadays, because of their resistance to fading upon exposure to light, water, and many chemicals. Among all the reported methods, photocatalysis is the most favorable one due to its high efficiency and energy saving [51,52,53]. In this sense, different types of COF-based catalysts have been developed for water decontamination.

Nitroaromatic compounds (NACs) are a group of industrial chemicals commonly used in the production of dyes, polymers, or pesticides. Their presence in the environment has led to the pollution of soil and groundwater. Moreover, they are extremely dangerous for human health, so it is essential to find a way for their degradation. Su et al. [54] developed a new COF-based catalyst for the reduction reaction of NACs into aromatic amines. Therefore, a high crystalline COF, TPHH-COF, was designed as the support for silver nanoparticles (Ag NPs), which have excellent catalytic activity in the degradation of these compounds. TPHH-COF possesses rich nitrogen and oxygen reactive sites that act as reactive functional sites to help improve the interactions and increase the metal-binding sites enhancing the activity of the material. The first organic pollutant studied was 4-nitrophenol (4-NP). The reduction of 4-NP with NaBH_4_ is thermodynamically advantageous, although kinetically limited without a suitable catalyst. It is a pseudo-first-order reaction and has an apparent rate constant (k_app_) of 0.022 s^−1^. The k_app_ increases with the amount of catalyst at 20 °C, and the catalyst maintained 97% of its initial catalytic capacity after six runs confirming its reusability. Five more NACs were selected; 2-nitrophenol (2-NP), 4-nitroaniline (4-NA), 1-butoxy-4-nitrobenzene, 4-nitrotoluene, and 4-nitrobenzene, to investigate the catalytic performance of the material. Results for 2-NP, 4-NA, 1-butoxy-4-nitrobenzene, 4-nitrotoluene and 4-nitrobenzene, showed k_app_ values of 0.0173 s^−1^, 0.0335 s^−1^, 0.0046 s^−1^, 0.0086 s^−1^, and 0.0079 s^−1^, respectively. 

**Figure 16:**
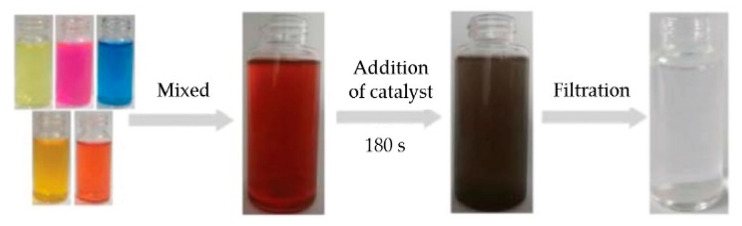
Reductive degradation and discoloration of a sample mixture of 4-nitrophenol and four different dyes in water catalyzed by Ag@TPHH-COF. Conditions of volume and concentration: 4-NP (10 mL, 5 × 10^−4^ mol L^−1^), RhB (10 mL, 5 × 10^−5^ mol L^−1^), MB (10 mL, 1 × 10^−4^ mol L^−1^), MO (10 mL, 2.5 × 10^−4^ mol L^−1^), and CR (10 mL, 5 × 10^−4^ mol L^−1^); catalyst mass = 5 mg); NaBH_4_ = 0.5 g. Reprinted with permission from [54]. Copyright (2019) The Royal Society of Chemistry.

The reduction of several dyes by Ag@TPHH-COF was also studied for their catalytic hydrogenation reduction. The organic dyes selected were RhB, MB, MO, and CR. Right after the catalyst is added, the color of the solution disappears quickly, showing that the reduction of the dyes occurs rapidly in the presence of Ag@TPHH-COF. Decolorization times of RhB, MB, MO, and CR were 270 s, 120 s, 240 s, and 600 s. The Ag@TPHH-COF catalyzes the hydrogenation reaction of -N=N- and -C=N- groups and destroys their conjugated structure. The values of k_app_ were 0.0127 s^−1^, 0.030 s^−1^, 0.0180 s^−1^, and 0.005 s^−1^, respectively. To finally determine the applicability of the catalyst for reduction reactions of a simulated wastewater sample, the group carried out the experiment mixing the four dyes and 4-NP (Figure 16). After adding NaBH_4_ and the catalyst and stirring for 3 min, the solution was filtered and found colorless.

**Figure 17:**
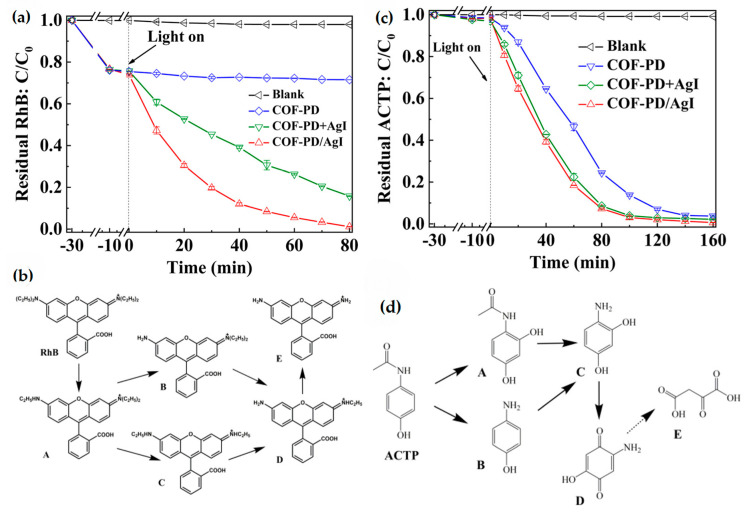
The photocatalytic degradation performance of RhB (**a**) and ACTP (**c**) by the materials under visible light irradiation; the VLD photocatalytic degradation mechanism of RhB (**b**) and ACTP (**d**) by COF-PD/AgI. Reprinted from [55]. Copyright (2020) with permission from Elsevier.

The photocatalyst fabricated by Tong Liu et al. [55] in 2020, based on an AgI modified COF, COF-PD/AgI, using 2,5-diaminopyridine and 1,3,5-triformylphloroglucinol as precursors, also demonstrated good efficiency in organic pollutants degradation. In this case, the reaction consisted of a visible light-driven (VLD) photocatalytic process. COFs present periodic π-conjugated aromatic units that make them good organic semiconductors to serve as the pathway for electrons (***e^−^***)-holes (***h***^+^) transport. Because it has been demonstrated before that heterojunction could promote the separation of photo-induced ***e^−^*** and ***h^+^*** in VLD photocatalysis, the authors modified the COF with AgI, to enhance the performance of the catalyst under visible light. AgI is ideal due to its good absorption of visible light and optimal band position. The synthesis starts with the obtention of COF-PD via solvothermal reaction, followed by co-precipitation of AgI (diameter of 5–20 nm) to obtain the COF-PD/AgI, and its performance towards organic pollutants degradation was tested for RhB and Acetaminophen (ACTP), which is an antipyretic drug that belongs to the pharmaceutical and personal care products family (PPCPs). RhB is completely removed by the COF-PD within 80 min (Figure 17a), being the pseudo-first kinetic constant 4.66 × 10^−2^ min^−1^, whereas under dark conditions, only around 25% of the dye is adsorbed. It confirms the improvement of the photocatalytic degradation of RhB, thanks to the heterojunction between the COF and AgI nanoparticles. The process proposed for the degradation of RhB is N-deethylation (Figure 17b). ACTP’s complete removal by COF-PD/AgI under visible light irradiation happens within 60 min (k = 3.34 × 10^−2^ min^−1^) (Figure 17c). Whereas under dark conditions, the material removes less than 2% of ACTP. In this case, the degradation process follows up a hydroxylation reaction (Figure 17d). The photocatalyst shows good performance within four cycles towards the two pollutants with a removal percentage of 97 and 99%, respectively.

Moreover, COF-PD/AgI photo-catalytically disinfects bacteria, such as *Escherichia coli* (E. coli), from wastewater. In this case, the photocatalytic mechanism followed up the viable cell density of *E. coli*, initially being 1.5 × 10^6^ CFU mL^−1^. SEM microscopy was used to observe *E. coli’s* membrane before and after its degradation. These experiments were also carried out under dark conditions showing that the COF-PD/AgI alone did not lead to bacteria disinfection; while carried out under visible light irradiation, the material thoroughly disinfects 1.5 × 10^6^ CFU mL^−1^ within 40 min.

Another typical chemical compound that can be accumulated in the human body, causing severe health effects, is Bisphenol A (BPA). BPA is used in the industry of paper and the synthesis of polymers. Again, the most promising method to approach the degradation of BPA in wastewater samples is AOPs. Based on the combination of MOFs and COFs with C_3_N_4_ active units to improve the photocatalytic activity, Wang et al. [56] have recently reported a new hybrid material, achieving an enhanced BPA degradation efficiency. The selected MOFs were the UiO-66-NH_2_ and MIL-101-NH_2_ with the COF TpMA, assembled to give the MIL-101-NH_2_@TpMA and UiO-66-NH_2_@TpMA photocatalysts. MOFs@COFs hybrids were employed as photocatalytic platforms coupling with sulfate radical (SO_4_^−^)-based AOPs (SR-AOPs). SO_4_^−·^ showed better oxidation ability than the hydroxyl radical (^·^OH) in degrading processes and can be produced via activation of peroxydisulfate (PS).

**Figure 18:**
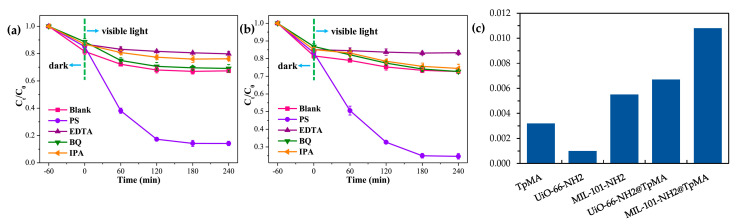
(**a**) Photocatalytic degradation of BPA using the catalysts under visible light irradiation (Conditions: catalyst = 5 mg, PS = 10 mg, pH = 5, volume = 20 mL and BPA = 50 mg L^−1^). (**b**) The pseudo-first-order kinetics for BPA degradation and (**c**) calculated K (min^−1^). Reprinted from [56]. Copyright (2019) with permission from Elsevier.

The functionalization of MOFs and COFs can turn these materials into promising photocatalysts, for example, introducing nitrogen-rich functional groups would improve the visible-light response of the COF. The catalysts will act as activators of PS for the degradation of BPA by using solar energy. The preparation of the hybrid composites was following a one-step hydrothermal method. For the prepared catalysts, an increase of the absorption was observed, attributed to the introduction of the C_3_N_4_ units, that act as visible-light active centers that facilitate the generation of photo-produced charge carriers. The results showed that the photocatalytic performance of the resultant MIL-101-NH_2_@TpMA and UiO-66-NH_2_@TpMA towards BPA (Figure 18a), were enhanced by the introduction of the C_3_N_4_ and the heterojunction formed at the interface with covalent bonds was beneficial for the separation and transfer of photogenerated electro-hole pairs. Pseudo-first-order kinetics show that the degradation rates of the catalysts for BPA are higher than the precursors (Figure 18b). The highest kinetic rate constant obtained belonged to the MIL-101-NH_2_@TpMA, with a value of 0.0108 min^−1^, and the highest degradation efficiency was achieved with a PS concentration of 20 mg, being 99% for the MIL-101-NH_2_@TpMA and 82% for the UiO-66-NH_2_@TpMA. Both catalysts were reusable for over five cycles, reaching a 83 and 69% degradation efficiency, respectively.

**Figure 19:**
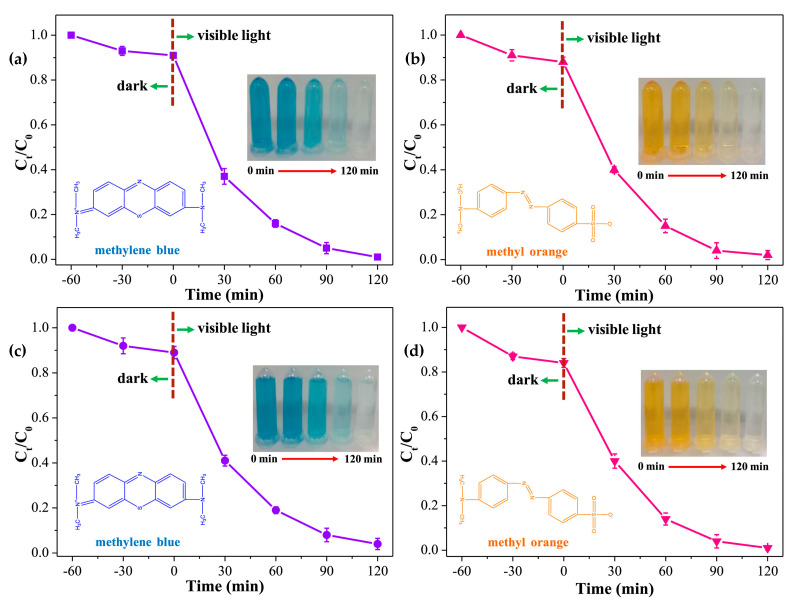
Photocatalytic degradation of MB (**a**) and MO (**b**) with MIL-101-NH_2_@TpMA under visible light irradiation; Photocatalytic degradation of MB (**c**) and MO (**d**) with UiO-66-NH_2_@TpMA under visible light irradiation. (Conditions: catalyst = 5 mg; PS, 10 mg; pH = 5; volume, 20 mL; MB/MO concentration = 20 mg L^−1^). Reprinted from [56]. Copyright (2019), with permission from Elsevier.

These hybrid photocatalysts also possessed good efficiency in dye degradation, especially towards oxidative degradation of MO and MB dyes after 120 min (Figure 19).

### 3.5. COFs for Capture and Detection

- Bisphenol A

**Figure 20:**
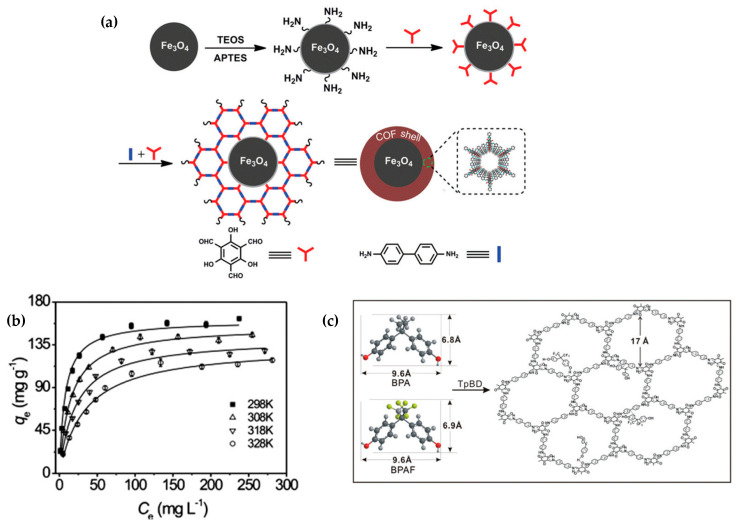
(**a**) Illustration of the monomer-mediated in situ growth strategy for the synthesis of core–shell Fe_3_O_4_@TpBD nanospheres. (**b**) Adsorption isotherms of BPA on Fe_3_O_4_@TpBD. (**c**) Illustration for the mechanism of π-π interaction and hydrogen bonding between BPA/BPAF and TpBD. Reprinted with permission from [57]. Copyright (2017) The Royal Society of Chemistry.

Many studies are reporting different materials based on the capture of BPA. In 2017, Li et al. [57] showed a procedure to obtain COF shells on magnetic nanospheres. Fe_3_O_4_ was selected as the super-paramagnetic core, while the porous COF TpBD (1,3,5-triformylphloroglucinol and benzidine, respectively) acted as the shell. The magnetic core-shell nanospheres (Fe_3_O_4_@TpBD) showed great potential for BPA and bisphenol AF (BPAF) removal from aqueous media samples. The synthesis relies on a monomer-mediated in situ growth approach (Figure 20a). Then, Fe_3_O_4_@TpBD nanospheres with a 65 nm COF shell thickness were studied on BPA adsorption and magnetic separation. The kinetics showed equilibrium for BPA was achieved within 5 min. It followed a pseudo-second-order kinetic, suggesting the adsorption was based on the adsorption capacity of the surface sites on Fe_3_O_4_@TpBD at equilibrium. The resulting maximum adsorption capacity of BPA was 160.6 mg g^−1^ (Figure 20b). There is a π–π interaction and hydrogen bonding effect between the carboxyl groups of the COF and the hydroxyl group in BPA, as the molecule goes through the channels of the COF (Figure 20c). Besides, BPA and BPAF can be desorbed from the magnetic core-shell with ethanol in 1 min.

**Figure 21:**
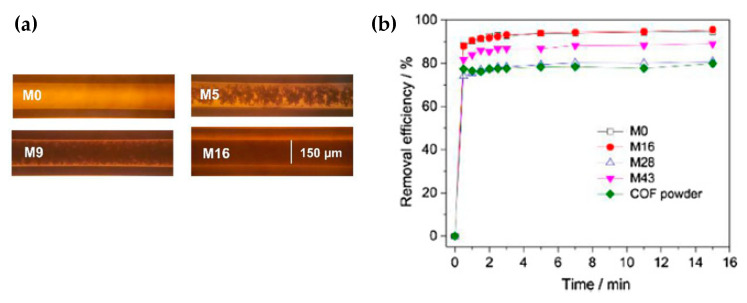
(**a**) Optical images of pre-polymerization mixtures inside capillary before the formation of the monoliths. (**b**) Removal efficiency of BPA by COF powder and monoliths. Reprinted from [48]. Copyright (2018), with permission from Elsevier.

Later on, in 2018, Liu et al. [48] prepared other monoliths varying the COF weight percentages from 0 to 43%. Monoliths were prepared inside capillaries of 150 μm diameter, modified with amino group layers on the inside to anchor the monoliths (Figure 21a). The COF capillary column retained BPA and other hydrophilic pollutants when the mobile phase was water and could be eluted with methanol. For M0, the removal efficiency reached 93.8% in 5 min, and for the COF powder, 77.4% in 0.5 min, for the rest of the monoliths, M16, M28, and M43, it took 3 min [48] (Figure 21b). The pseudo-second-order rate constants (K2) and the adsorbate capacity are presented in Table 1.

**Figure 22:**
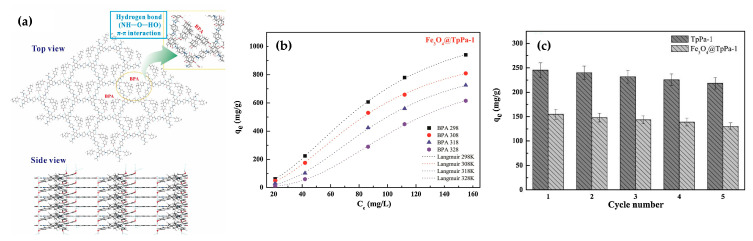
(**a**) The lowest energy adsorption configuration of BPA adsorbed onto the surface of TpPa-1. (**b**) Adsorption–desorption cycles of BPA onto TpPa-1 and Fe_3_O_4_@TpPa-1. (**c**) Fitting curves for BPA adsorption onto TpPa-1 and Fe_3_O_4_@TpPa-1 using Langmuir model. Reprinted from [58]. Copyright (2020), with permission from Elsevier.

In 2020, Hu et al. [58] fabricated a magnetic COF based on the Tp-series. The product, Fe_3_O_4_@TpPa-1, which showed outstanding performance in the extraction of BPA in aqueous samples, was synthesized using a one-step hydrothermal method. The Fe_3_O_4_@TpPa-1 NPs with an average diameter between 80 and 100 nm had a core–shell structure similar to the one mentioned before fabricated by Yan et al. [57]; for this material, TpPa-1 thickness was around 10 nm. Again, the hydrogen bonding effect and the π–π interaction between the functional groups of the COF and BPA were the main factors in the adsorption process of BPA by Fe_3_O_4_@TpPa-1, aside from porosity (Figure 22a). The adsorption isotherms were obtained to suit the Langmuir model with a resulting maximum adsorption capacity of 1424.27 and 1220.97 mg g^−1^ BPA at 298 K, for the TpPa-1 and Fe_3_O_4_@TpPa-1 [58], respectively (Figure 22b). Besides its large capacity, the material also exhibited good performance in five consecutive adsorption-desorption cycles, with a low recovery decrease (Figure 22c).

- Hg(II) detection and removal

Hg(II) is one of the most toxic heavy metal ions present in aqueous media. Its detection and removal from the environment to avoid its bioaccumulation has been studied for decades.

In 2017, Merí-Bofí et al. [59] designed a thiol grafted imine-based COF (TPB-DMTP-COF-SH) by using as starting precursor an imine-linked COF with reactive ethynyl groups on the walls of 1D pores as a platform to incorporate triazole and thiol groups on surface pores via click reaction. The post-synthesis modification of the precursor with triazole and thiol groups provided channels with flexible arms for Hg(II) capture due to the high affinity of sulfur atoms and the high coordinative capacity of triazole groups for this heavy metal ion. This new material presents the largest adsorption capacity of all the COFs mentioned (Table 2), of 4395 mg g^−1^ and a retention percentage of 99.98% in water within 2 minutes for Hg(II).

Later, in 2019, Guo et al. [60] fabricated a carboxy-functionalized COF (COOH@COF) to remove not only Hg(II), but Pb(II) too. The carboxy function was grafted to the COF *via* a click reaction. SEM images of the synthesized COOH@COF showed that the material possesses a thorn ball structure, with 90 nm diameter nanowires. The best COF performance was obtained at pH 6.0 and 25 °C. Its maximum adsorption capacity for Hg(II) was 99.1 mg g^−1^, and the removal percentage above 90% in the prepared solutions was maintained even after 20 cycles of use.

**Figure 23:**
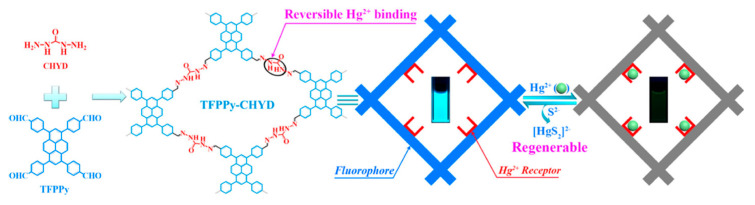
Synthesis of the preparation of TFPPy-CHYD COF, its fluorescence detection and Hg(II) adsorption, and regeneration by adding Na_2_S. Reprinted with permission from [61]. Copyright (2019) American Chemical Society.

In the last year, more Hg(II) selective COFs have been reported. Qiu’s group introduced a regenerable carbohydrazide-linked fluorescent COF for the detection and adsorption of this metal [61]. The material, TFPPy-CHYD COF, combines a pyrene-based building block with a carbohydrazide (CHYD) linker (Figure 23). This leads to luminescence enhancement and the fluorescence response of the material; when Hg(II) is present, it can be observed with a UV lamp and provides reversible binding sites for Hg(II). The obtained material efficiently removes mercury, Hg^0^ and Hg(II), from both water and air, and the adsorption capacities were 232 and 758 mg g^−1^, respectively [61]. Around 99% of Hg(II) was removed within 5 min, reducing its concentration from 10 ppm to 2 ppb, which is under the limit for drinking water. Besides, the material can be reused within 6 cycles without losing sensitivity or rate removal by adding 10 equiv of Na_2_S solution.

In the same year, Wang et al. [62], reported the removal of mercury from acidic wastewater using a COF with Ag NPs anchored in it via one-step solution infiltration method. The obtained material, an Ag NPs@COF composite, is based on the COF-LZU1, which can be synthesized under ambient conditions and is stable in various solvents. The adsorption capacity of the composite was 113 mg g^−1^, which is much higher than that for the pristine COF, 24 mg g^−1^. Around 99% of the Hg(II) was removed from solutions at different pH values, pH = 1, 3, 5, 7. ICP-AES showed Ag leaching (3%) occurred in the two lowest pH values. Mercury captured could be easily regenerated by thermal decomposition at moderate temperature (200 °C), and the Ag NPs@COF-LZU1 exhibited good performance upon five cycles without significant removal percentage loss.

Finally, Afshari et al. [63] fabricated a triazine ring-based COF (T-COF) superadsorbent for Hg(II) removal. The group also developed a portable device for *in situ* detection of trace amounts of Hg(II). The colorimeter system had a linear response in the concentration range of 0.25–15 mg L^−1^, and the detection limit is 0.063 mg L^−1^ of Hg(II). The calculated adsorption capacity of the superadsorbent was 1826 mg g^−1^ of Hg(II) ions at ambient temperature and pH = 5, and the adsorbent removal efficiency was excellent up to three cycles. The large adsorption capacity of the T-COF can be due to the interaction between Hg(II) ions and the nitrogen atoms present on the material’s surface by electrostatic attraction between the oxygen atoms and the ions, and also by ion–π interactions with the double bonds. This material, together with the sensing device, makes this method a good option to detect and determine Hg(II) ions at an industrial scale.

- Pb(II) detection and removal

Besides Hg (II), another toxic heavy metal ion present in the environment that can cause several health problems after its bioaccumulation is lead. It is used in a great variety of industries, such as the mining industry, the smelting industry, and many others. A handful of methods have been investigated for its removal from water, but adsorption has proven to be one of the most promising ones.

The carboxy-functionalized COF (COOH@COF), reported by Guo et al. in 2019 also showed good performance towards Pb(II) removal from water. The COF had high selectivity for Pb(II) ions, exhibiting a larger adsorption capacity for Pb(II) than Hg(II), with a value of 123.9 mg g^−1^ and a removal percentage above 90% [60]. As it did with Hg(II) ions, the material’s regeneration allows its usage up to 20 cycles, maintaining the same adsorption capacity for Pb(II).

The same year, Xu et al. [64] reported a COF with triazine (Tz) and hydroxyl (OH) bifunctional groups, denominated COF-Tz-OH, which served as a scaffold for Pb(II) ions. The calculated adsorption capacity was 476 mg g^−1^, exhibiting 99% removal efficiency within 5 min. The large capacity is related to the amount of available chelating sites from the bifunctional groups present in the mesopores of the structure. Furthermore, the material can be reused, maintaining more than 90% removal efficiency after five cycles.

In 2020, Cao et al. [65] synthesized a new sulfhydryl functionalized COF (COF-SH), by a mild solvothermal solution-suspension method, for selective Pb(II) removal in water. The sulfhydryl groups in the structure of the COF acted as active adsorption sites for Pb(II) ions capture. The introduction of SH groups on the structure changed the electrostatic potential on the surface, becoming more electronegative and leading to better adsorption of lead ions. The adsorption capacity for this material was 239 mg g^−1^ of Pb(II), achieving adsorption equilibrium within 48 h, with a similar removal percentage (>90%) in the presence of other metal ions and that with just water contaminated with Pb(II).

- Non-steroidal anti-inflammatory drugs

Non-steroidal anti-inflammatory drugs (NSAIDs) are a class of drugs used as analgesics to treat and reduce pain and, in higher doses, inflammation. Because of their massive production, these compounds are starting to accumulate in many sources such as rivers or groundwaters, leading to significant environmental and health problems.

Last year, Liang et al. [66] reported a COF with high selectivity and significant adsorption for this type of contaminants from wastewater. Two COFs (COF-NO_2_ and COF-NH_2_) were synthesized, and three NSAIDs were specifically selected to study: Ketoprofen (KTP), Ibuprofen (IBP), and Naproxen (NPX). The COF precursor was the TPB-DMTP-COF for later incorporation of nitro groups by post-synthetic modification, followed by their reduction to amino groups. The maximum adsorption capacity of the COF-NO_2_ for KTP, IBP, and NPX was 70, 94, and 80 mg g^−1^, respectively. The COF-NH_2_ exhibited less adsorption capacity but more selectivity for KTP than the other two contaminants, which did not happen with the first COF. The adsorption capacities were 33, 18, and 16 mg g^−1^ of KTP, IBP, and NPX, respectively. For the COF-NO_2_ the interaction between the COF and the organic contaminant structure is through hydrogen bonds. On the other hand, the significantly higher selectivity of COF-NH_2_ for KTP than the other two can be due to the interactions between the -NH_2_ groups of the COF and KTP, including the formation of hydrogen bonds, acid–base interactions, and strong force C=N bonds with the carbonyl group. In contrast, the IBP and NPX could not form the last one.

- Dye compounds

In 2017, Zhu et al. [67] synthesized a triazine-functionalized polyimide COF that exhibited large adsorption of methylene blue (MB). Two COFs were synthesized, the macroporous TS-COF-1, a triazine-polyimide-COF obtained from PMDA and TAPT building blocks, and the microporous TS-COF-2, a triazine-Schiff base-COF with TP and TAPT as building blocks. The maximum adsorption capacity of TS-COF-1 is 1691 mg g^−1^ of MB, one of the largest reported to date. For the microporous TS-COF-2 the adsorption capacity was smaller, with a value of 377 mg g^−1^ MB. This is due to the size of the pores in the COF structure, 31 Å, and 11 Å, for the meso- and microporous COFs, respectively, as MB’s size is 13.4 Å × 5.0 Å × 4.2 Å, it was easier for the molecule to enter and diffuse through TS-COF-1. The weak interactions between MBs and the COF allowed the regeneration of the material, and the adsorption percentage was maintained after three cycles. The material was also studied to remove two more dyes, including RhB and CR, obtaining a maximum adsorption capacity of 625 and 319 mg g^−1^, respectively. Two more organic pollutants, 4-nitrophenol and 3-nitrophenol, were studied, and the adsorption capacities obtained were 369 and 424 mg g^−1^, respectively.

- Detection and removal of antibiotics

Nitrofurans (NFs) are a family of bacterial antibiotics with a defined structure composed of a nitrofuran group with different side chains. They are commonly used in medical treatment, but also in the breeding industry, although they are not authorized by the EU in food-producing animals. Because their accumulation in water can cause several health problems for humans, their detection and removal are crucial.

**Figure 24:**
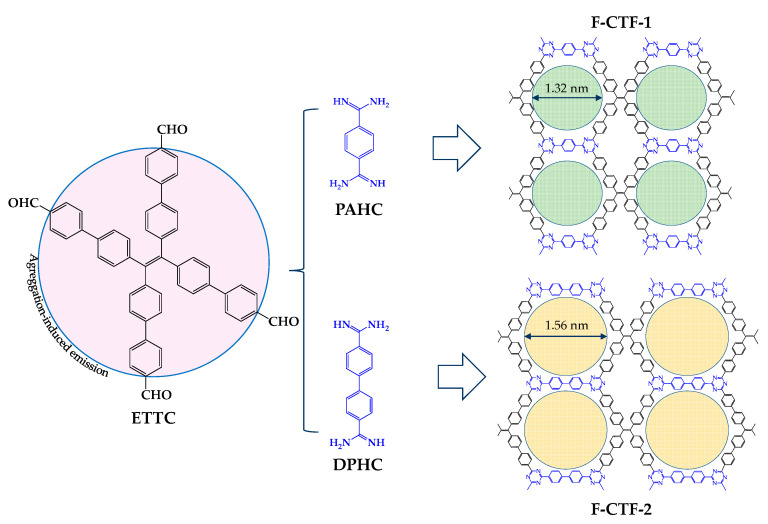
Synthesis of 2D porous F-CTFs by the condensation reaction of AIE aldehyde monomer and the two different amidine (PAHC and DPHC) under mild conditions.

There are many methods to detect these compounds, including high-performance liquid chromatography or gas chromatography–mass spectrometry. However, efforts are still being made to develop an efficient and rapid detection technique. Tang et al. [68] have recently reported two covalent triazine frameworks (CTFs) for the simultaneous detection and removal of three of these antibiotics from water: nitrofurazone (NZF), nitrofurantoin (NFT), and furazolidone (FZD). The 2D CTF nanosheets were based on aggregation-induced emission luminogens (AIEgens), which exhibited high luminescence properties in the solid-state. Two CTFs were synthesized by condensation reaction between the building blocks, 4′,4‴,4″″′,4‴‴′-(ethene-1,1,2,2-tetrayl)tetrakis(([1,1′-biphenyl]-4-carbaldehyde)) (ETTC) monomer with AIE behavior, and phenamidine hydrochloride (PAHC) and diphenamidine hydrochloride (DPHC), to give ultrathin lamellar layers F-CTF-1 and F-CTF-2 (Figure 24), with the thickness of 1.48 and 1.06 nm. The results proved that rigidifying AIEgen in 2D CTF nanosheets could modify the electronic transition energies and increase the luminous efficacy. The group tested over 12 different antibiotics, determining that NZF, NFT, and FZD were the ones that increased the quenching efficiencies the most for both CTFs. The calculated limits of detection (LODs) towards the antibiotics for the F-CTF-1 were 4.97, 8.08, and 13.35 ppb for NZF, NFT, and NZD, respectively. Additionally, the F-CTF-2 was 1.75, 2.21, and 3.86 ppb. Finally, maximum adsorption capacities of F-CTF-1 and -2 were 351 and 298 mg g^−1^ for NZF, 240 and 200 mg g^−1^ for NFT, and 196 and 154 mg g^−1^ for FZD, respectively, at pH 6. The mechanism proposed is that the N atoms of triazine rings in the COFs interact strongly with the adsorbates as the preferential adsorption sites. Additionally, both CTFs show recyclability, recovering their initial adsorption capacity over five consecutive cycles.

**Table 2:** Comparative adsorption performance of the mentioned COFs and several benchmark adsorbents towards each adsorbate.

COFs	Adsorbate	Capacity (mg g^−1^)	Active Sites	Reference
Fe_3_O_4_@TpBD	BPA	160	Carboxyl groups	[57]
Hierarchically porous monolith-COF (M16)	BPA	22	Aldehyde and hydrazine groups	[48]
Fe_3_O_4_@TpPa-1	BPA	1220	Amine and carbonyl groups	[58]
TPB-DMTP-COF-SH	Hg(II)	4395	Thiol and triazole functional groups	[59]
COOH@COF	Hg(II)	99	Carboxyl and thioether groups	[60]
TFPPy-CHYD COF	Hg(II)	758	Secondary amine group	[61]
AgNPs@COF-LZU1	Hg(II)	113	-	[62]
T-COF	Hg(II)	1826	Nitrogen and oxygen atoms	[63]
COOH@COF	Pb(II)	124	Carboxyl and thioether groups	[60]
COF-Tz-OH	Pb(II)	476	Triazine and hydroxyl groups	[64]
COF-SH	Pb(II)	239	Sulfhydryl grupos	[65]
COF-NO_2_	KTP IBP NPX	70 94 80	Amino groups	[66]
COF-NH_2_	KTP IBP NPX	33 18 16	Amino groups	[66]
TS-COF-1	MB RhB CR 4-NP 3-NP	1691 625 319 369 424	Triazine groups	[67]
TS-COF-2	MB	377	Triazine groups	[67]
F-CTF-1	NZF NFT FZD	351 240 196	Triazine groups	[68]
F-CTF-1	NZF NFT FZD	298 200 154	Triazine groups	[68]
MIL-53 (Cr) (MOF)	BPA	421	π-π interaction, hydrogen bonding	[69]
ED-MIL-101(Cr) (MOF)	NPX	154	Acid-base interaction	[70]
NH_2_-MIL-101(Al) (MOF)	4-NP MB	193 1409	Hydrogen bonding Electrostatic interaction	[71,72]
MnO_2_ nanotubes@rGO (MNGH)	Pb(II)	356	-	[73]
Na^+^ modified rGO-Fe_3_O_4_(SMGI)	Pb(II)	1666	-	[74]

## 4. Conclusions

Water is a vital human resource that needs to be optimized to avoid waste. The re-use of treated wastewater offers considerable social, financial, and environmental advantages. Moreover, when compared to different sources of water supply such as desalination, water reuse often turns out to require lower economic costs and energy, therefore causing a reduction of greenhouse gas emissions. Currently, membrane technology is the favorite choice for reclaiming water from different wastewater streams for water reuse. Modern membranes based on organic polymers show dense and amorphous structures suffering from a trade-off between permeability and selectivity [75].

Over the last few years, the controlled assembly of molecules has allowed the preparation of crystalline materials with high porosity, uniform and tunable pore size, and adjustable surface properties with well-defined tuned porous structures that can be molecularly designed for specific applications [76]. They are appealing alternatives in the field of advanced separation. In this context, only MOFs and COFs allow polymeric atomically precise structures. The number of potential applications is enormous, and some of the most relevant are those concerning energy, gas storage and/or separation, or water treatment. Many of these applications require membranes, e.g., gas separation and water treatment. MOFs and COFs are excellent for membrane materials because they present superior capability of acting as selective barriers to allow the transportation of chemical species (i.e., molecules or ions). In particular, COFs based on imine are more thermally and chemically robust than MOFs [77]. Therefore, COF membranes seem to be excellent candidates for many applications.

Hence, it remains an enormous challenge to solve the trade-off issue to obtain high permeability and high selectivity in the same membrane. Motivated by the chances offered by COFs, continuous and defect-free COF membranes have enormous potential to meet the aim of precise sieving and, at the same time, break the trade-off problem. However, even though continuous and crystalline COF membranes are challenging, different strategies have already been developed to prepare several membranes based on COFs.

Revising the current state-of-the-art COF membranes fabrication in this article suggests that this is a relatively new field. We have identified two fundamental strategies have already been followed for COF membrane fabrication: (i) those based on the use of porous support, and (ii) free-standing COF membranes. The first set collects from the formation of COFs on different macroporous organic and inorganic supports to the preparation of mixed matrix membranes in which the CONs act as fillers. In our opinion, the emergence of COF nanosheets has brought an alternative for the preparation of ultrathin laminated films with tunable thicknesses from hundreds of nanometers to few nanometers. These CON membranes let the flow media pass through the COF films directly rather than along the in-plane CONs entirely. This is because the transport channels are located perpendicular to the transport direction, reducing the length and tortuosity of transport pathways. Most previous research works have focused on fabricating self-standing COF membranes without supporting substrates; however, they need a thickness of hundreds of micrometers to ensure mechanical strength is sufficient for practical separations, in which application of pressure is needed. Indeed, the mechanical properties of COFs is a critical issue to be developed [13]. However, the thick separation layer significantly increases the mass transport resistance and decreases the permeation flux, causing a harsh barrier to overcome the trade-off issue among permeability and selectivity.

The research already carried out in this field shows great potential for the use of well-designed COF membranes since it brings significant improvements as a consequence of the *ad hoc* pore size and functionalization, which make them suitable for capture of specific contaminating agents such as toxic metal ions and organic molecules. Therefore, it is highly needed for future practical COF membrane applications to develop advanced approaches to produce mechanically robust and ultrathin defect-free COF membranes, particularly from sub-1 mm-thick to thin molecular membranes. Additionally, for industrial applications of thin COF membranes, some key issues have to be encountered as long/medium-term thermal and chemical stability, even under acidic/basic conditions, and antifouling ability during the water treatment.

Finally, it is worth mentioning the significant progress made during the last years in nanoscience and nanofabrication, with the discoveries of different alternative 2D graphene materials as graphene oxide, boron nitride, transition metal dichalcogenides, MXene; the use of COFs has added new perspectives into ultrathin separation membranes [78]. Compared to other non-porous 2D thin-films, a defect-free and large-scale molecular thick COF film would show utmost potential towards ultimate separation due to the shortest transport pathways. Combining control of thickness and mechanical properties is still quite challenged; however, we firmly believe that alternative novel COF membranes will improve the current membrane technology, providing new solutions to solve these practical problems.

Very recently, the shaping of imine-based COFs into monoliths has opened new perspectives in water applications for decontamination of organic solvents and dyes [13,15,16].

Finally, other important issues with implementing COFs for large-scale applications still need development. For instance, COFs activation is fundamental for material preparations and shaping (e.g., dense monoliths or monolith aerogels) and a crucial point for some applications (e.g., gas sorption and/or storage, catalysis). Obviously, it has to be considered for scale-up applications, since the use of supercritical CO_2_ is not the best alternative. However, very recently, some simple COF activations have reported the use of gas flow for COF activation with excellent results that could represent an industrial solution. Anyway, some of these important aspects still need further development [79]. However, COFs for water treatment do not typically require activation, but rather solvent exchange. It simplified this complex aspect, since COF activation will not be necessary.

In summary, we believe that this review can provide potential guidance for the current state of the art and perspectives on the preparation and uses of COF membranes, and help accomplish ultimate separation and motivate creative new design in this research.

## Figures and Tables

**Table 1:** Pseudo-second-order rate constant (K_2_) and Qe of the synthesized materials.

Sample	K_2_/(g mg^−1^ min^−1^)	Qe/(mg g^−1^)
M0	0.82	21.7
M16	0.58	21.9
M28	0.65	18.5
M43	0.63	20.4
COF powder	0.90	18.1

## References

[1] Lohse M.S., Bein T. (2018). Covalent Organic Frameworks: Structures, Synthesis, and Applications. Adv. Funct. Mater..

[2] Ongari D., Yakutovich A.V., Talirz L., Smit B. (2020). Building a consistent and reproducible database for adsorp-tion evaluation in Covalent-Organic Frameworks. Mater. Cloud Arch..

[3] Segura J.L., Royuela S., Mar Ramos M. (2019). Post-synthetic modification of covalent organic frameworks. Chem. Soc. Rev..

[4] Waller P.J., Gándara F., Yaghi O.M. (2015). Chemistry of Covalent Organic Frameworks. Acc. Chem. Res..

[5] Xia Z., Zhao Y., Darling S.B. (2021). Covalent Organic Frameworks for Water Treatment. Adv. Mater. Interfaces.

[6] Côté A.P., Benin A.I., Ockwig N.W., O’Keeffe M., Matzger A.J., Yaghi O.M. (2005). Porous, Crystalline, Covalent Organic Frameworks. Science.

[7] Côté A.P., El-Kaderi H.M., Furukawa H., Hunt J.R., Yaghi O.M. (2007). Reticular synthesis of microporous and mesoporous 2D covalent organic frameworks. J. Am. Chem. Soc..

[8] Ding S.-Y., Gao J., Wang Q., Zhang Y., Song W.-G., Su C.-Y., Wang W. (2011). Construction of Covalent Organic Framework for Catalysis: Pd/COF-LZU1 in Suzuki–Miyaura Coupling Reaction. J. Am. Chem. Soc..

[9] Uribe-Romo F.J., Doonan C.J., Furukawa H., Oisaki K., Yaghi O.M. (2011). Crystalline covalent organic frameworks with hydrazone linkages. J. Am. Chem. Soc..

[10] Kandambeth S., Mallick A., Lukose B., Mane M.V., Heine T., Banerjee R. (2012). Construction of Crystalline 2D Covalent Organic Frameworks with Remarkable Chemical (Acid/Base) Stability via a Combined Reversible and Irreversible Route. J. Am. Chem. Soc..

[11] Li Y., Wang C., Ma S., Zhang H., Ou J., Wei Y., Ye M. (2019). Fabrication of Hydrazone-Linked Covalent Organic Frameworks Using Alkyl Amine as Building Block for High Adsorption Capacity of Metal Ions. ACS Appl. Mater. Interfaces.

[12] Kandambeth S., Dey K., Banerjee R. (2019). Covalent Organic Frameworks: Chemistry beyond the Structure. J. Am. Chem. Soc..

[13] Carrington M., Rampal N., Madden D., O’Nolan D., Casati N.P.M., Divitini G., Cepitis R., Ilan J.M., Camur C., Silvestre-Albero J. (2021). Sol-Gel Processing of a Covalent Organic Framework for the Generation of Hierarchically Porous Monolithic Adsorbents. ChemRxiv.

[14] Rodríguez-San-Miguel D., Zamora F. (2019). Processing of covalent organic frameworks: An ingredient for a material to succeed. Chem. Soc. Rev..

[15] Martín-Illán J.Á., Rodríguez-San-Miguel D., Castillo O., Beobide G., Perez-Carvajal J., Imaz I., Maspoch D., Zamora F. (2021). Macroscopic Ultralight Aerogel Monoliths of Imine-based Covalent Organic Frameworks. Angew. Chem. Int. Ed..

[16] Zhu D., Zhu Y., Yan Q., Barnes M., Liu F., Yu P., Tseng C.-P., Tjahjono N., Huang P.-C., Rahman M.M. (2021). Pure Crystalline Covalent Organic Framework Aerogels. Chem. Mater..

[17] Yan T., Lan Y., Tong M., Zhong C. (2019). Screening and Design of Covalent Organic Framework Membranes for CO2/CH4 Separation. ACS Sustain. Chem. Eng..

[18] Duan K., Wang J., Zhang Y., Liu J. (2019). Covalent organic frameworks (COFs) functionalized mixed matrix membrane for effective CO2/N2 separation. J. Memb. Sci..

[19] Cao X., Qiao Z., Wang Z., Zhao S., Li P., Wang J., Wang S. (2016). Enhanced performance of mixed matrix membrane by incorporating a highly compatible covalent organic framework into poly(vinylamine) for hydrogen purification. Int. J. Hydrogen Energy.

[20] Fan H., Mundstock A., Feldhoff A., Knebel A., Gu J., Meng H., Caro J. (2018). Covalent Organic Framework-Covalent Organic Framework Bilayer Membranes for Highly Selective Gas Separation. J. Am. Chem. Soc..

[21] Yin Y., Li Z., Yang X., Cao L., Wang C., Zhang B., Wu H., Jiang Z. (2016). Enhanced proton conductivity of Nafion composite membrane by incorporating phosphoric acid-loaded covalent organic framework. J. Power Sources.

[22] Li Y., Wu H., Yin Y., Cao L., He X., Shi B., Li J., Xu M., Jiang Z. (2018). Fabrication of Nafion/zwitterion-functionalized covalent organic framework composite membranes with improved proton conductivity. J. Memb. Sci..

[23] Wang Z., Zhang S., Chen Y., Zhang Z., Ma S. (2020). Covalent organic frameworks for separation applications. Chem. Soc. Rev..

[24] Yuan S., Li X., Zhu J., Zhang G., Van Puyvelde P., Van der Bruggen B. (2019). Covalent organic frameworks for membrane separation. Chem. Soc. Rev..

[25] Fang M., Montoro C., Semsarilar M. (2020). Metal and covalent organic frameworks for membrane applications. Membranes.

[26] Fan H., Gu J., Meng H., Knebel A., Caro J. (2018). High-Flux Membranes Based on the Covalent Organic Framework COF-LZU1 for Selective Dye Separation by Nanofiltration. Angew. Chem.-Int. Ed..

[27] Hao D., Zhang J., Lu H., Leng W., Ge R., Dai X., Gao Y. (2014). Fabrication of a COF-5 membrane on a functionalized α-Al 2O3 ceramic support using a microwave irradiation method. Chem. Commun..

[28] Wang Z., Si Z., Cai D., Li G., Li S., Qin P. (2020). Synthesis of stable COF-300 nanofiltration membrane via in situ growth with ultrahigh flux for selective dye separation. J. Memb. Sci..

[29] Pan F., Guo W., Su Y., Khan N.A., Yang H., Jiang Z. (2019). Direct growth of covalent organic framework nanofiltration membranes on modified porous substrates for dyes separation. Sep. Purif. Technol..

[30] Liu C., Jiang Y., Nalaparaju A., Jiang J., Huang A. (2019). Post-synthesis of a covalent organic framework nanofiltration membrane for highly efficient water treatment. J. Mater. Chem. A.

[31] Wang R., Shi X., Zhang Z., Xiao A., Sun S., Cui Z., Wang Y. (2019). Unidirectional di ff usion synthesis of covalent organic frameworks ( COFs ) on polymeric substrates for dye separation. J. Memb. Sci..

[32] Wang R., Wei M., Wang Y. (2020). Secondary growth of covalent organic frameworks (COFs) on porous substrates for fast desalination. J. Memb. Sci..

[33] Kandambeth S., Biswal B.P., Chaudhari H.D., Rout K.C., H. S.K., Mitra S., Karak S., Das A., Mukherjee R., Kharul U.K. (2017). Selective Molecular Sieving in Self-Standing Porous Covalent-Organic-Framework Membranes. Adv. Mater..

[34] Jiang Z., Livingston A.G., Santanu K. (2015). Sub-10 nm polyamide nanofilms with ultrafast solvent transport for molecular separation. Science.

[35] Jimenez-Solomon M.F., Song Q., Jelfs K.E., Munoz-Ibanez M., Livingston A.G. (2016). Polymer nanofilms with enhanced microporosity by interfacial polymerization. Nat. Mater..

[36] Dey K., Pal M., Rout K.C., Kunjattu S.S., Das A., Mukherjee R., Kharul U.K., Banerjee R. (2017). Selective Molecular Separation by Interfacially Crystallized Covalent Organic Framework Thin Films. J. Am. Chem. Soc..

[37] Matsumoto M., Valentino L., Stiehl G.M., Balch H.B., Corcos A.R., Wang F., Ralph D.C., Mariñas B.J., Dichtel W.R. (2018). Lewis-Acid-Catalyzed Interfacial Polymerization of Covalent Organic Framework Films. Chem.

[38] Valentino L., Matsumoto M., Dichtel W.R., Marinas B.J. (2017). Development and Performance Characterization of a Polyimine Covalent Organic Framework Thin-Film Composite Nanofiltration Membrane. Environ. Sci. Technol..

[39] Wang R., Shi X., Xiao A., Zhou W., Wang Y. (2018). Interfacial polymerization of covalent organic frameworks (COFs) on polymeric substrates for molecular separations. J. Memb. Sci..

[40] Su Y.Y., Yan X., Chen Y., Guo X.J., Chen X.F., Lang W.Z. (2021). Facile fabrication of COF-LZU1/PES composite membrane via interfacial polymerization on microfiltration substrate for dye/salt separation. J. Memb. Sci..

[41] Shinde D.B., Sheng G., Li X., Ostwal M., Emwas A.-H., Huang K.-W., Lai Z. (2018). Crystalline 2D Covalent Organic Framework Membranes for High-Flux Organic Solvent Nanofiltration. J. Am. Chem. Soc..

[42] Yang H., Yang L., Wang H., Xu Z., Zhao Y., Luo Y., Nasir N., Song Y., Wu H., Pan F. (2019). Covalent organic framework membranes through a mixed-dimensional assembly for molecular separations. Nat. Commun..

[43] Wang C., Li Z., Chen J., Li Z., Yin Y., Cao L., Zhong Y., Wu H. (2017). Covalent organic framework modified polyamide nanofiltration membrane with enhanced performance for desalination. J. Memb. Sci..

[44] Khan N.A., Khan N.A., Khan N.A., Yuan J., Yuan J., Wu H., Wu H., Wu H., Huang T., Huang T. (2020). Covalent Organic Framework Nanosheets as Reactive Fillers to Fabricate Free-Standing Polyamide Membranes for Efficient Desalination. ACS Appl. Mater. Interfaces.

[45] Xu L., Shan B., Gao C., Xu J. (2020). Multifunctional thin-film nanocomposite membranes comprising covalent organic nanosheets with high crystallinity for efficient reverse osmosis desalination. J. Memb. Sci..

[46] Wang H., Wang H., Jiang H., Sheng A., Wei Z., Li Y., Wu C., Li H. (2020). Positively Charged Polysulfonamide Nanocomposite Membranes Incorporating Hydrophilic Triazine-Structured COFs for Highly Efficient Nanofiltration. ACS Appl. Nano Mater..

[47] Wu C., Wang X., Zhu T., Li P., Xia S. (2020). Covalent organic frameworks embedded membrane via acetic-acid-catalyzed interfacial polymerization for dyes separation: Enhanced permeability and selectivity. Chemosphere.

[48] Liu Z., Wang H., Ou J., Chen L., Ye M. (2018). Construction of hierarchically porous monoliths from covalent organic frameworks (COFs) and their application for bisphenol A removal. J. Hazard. Mater..

[49] Li Y., Wu Q., Guo X., Zhang M., Chen B., Wei G., Li X., Li X., Li S., Ma L. (2020). Laminated self-standing covalent organic framework membrane with uniformly distributed subnanopores for ionic and molecular sieving. Nat. Commun..

[50] Yuan J., Wu M., Wu H., Liu Y., You X., Zhang R., Su Y., Yang H., Jiang Z. (2019). Covalent organic framework-modulated interfacial polymerization for ultrathin desalination membranes. J. Mater. Chem. A.

[51] Li N., Du J., Wu D., Liu J., Li N., Sun Z., Li G., Wu Y. (2018). Recent advances in facile synthesis and applications of covalent organic framework materials as superior adsorbents in sample pretreatment. TrAC-Trends Anal. Chem..

[52] Liang J., Liu F., Li M., Liu W., Tong M. (2018). Facile synthesis of magnetic Fe3O4@BiOI@AgI for water decontamination with visible light irradiation: Different mechanisms for different organic pollutants degradation and bacterial disinfection. Water Res..

[53] Hoffmann M.R., Martin S.T., Choi W., Bahnemann D.W. (1995). Environmental Applications of Semiconductor Photocatalysis. Chem. Rev..

[54] Wang R.L., Li D.P., Wang L.J., Zhang X., Zhou Z.Y., Mu J.L., Su Z.M. (2019). The preparation of new covalent organic framework embedded with silver nanoparticles and its applications in degradation of organic pollutants from waste water. Dalt. Trans..

[55] Liu F., Nie C., Dong Q., Ma Z., Liu W., Tong M. (2020). AgI modified covalent organic frameworks for effective bacterial disinfection and organic pollutant degradation under visible light irradiation. J. Hazard. Mater..

[56] Lv S.W., Liu J.M., Li C.Y., Zhao N., Wang Z.H., Wang S. (2020). Two novel MOFs@COFs hybrid-based photocatalytic platforms coupling with sulfate radical-involved advanced oxidation processes for enhanced degradation of bisphenol A. Chemosphere.

[57] Li Y., Yang C.X., Yan X.P. (2017). Controllable preparation of core-shell magnetic covalent-organic framework nanospheres for efficient adsorption and removal of bisphenols in aqueous solution. Chem. Commun..

[58] Zhong X., Lu Z., Liang W., Hu B. (2020). The magnetic covalent organic framework as a platform for high-performance extraction of Cr(VI) and bisphenol a from aqueous solution. J. Hazard. Mater..

[59] Merí-Bofí L., Royuela S., Zamora F., Ruiz-González M.L., Segura J.L., Muñoz-Olivas R., Mancheño M.J. (2017). Thiol grafted imine-based covalent organic frameworks for water remediation through selective removal of Hg(II). J. Mater. Chem. A.

[60] Lu X.F., Ji W.H., Yuan L., Yu S., Guo D.S. (2019). Preparation of Carboxy-Functionalized Covalent Organic Framework for Efficient Removal of Hg2+ and Pb2+ from Water. Ind. Eng. Chem. Res..

[61] Cui W.R., Jiang W., Zhang C.R., Liang R.P., Liu J., Qiu J.D. (2020). Regenerable Carbohydrazide-Linked Fluorescent Covalent Organic Frameworks for Ultrasensitive Detection and Removal of Mercury. ACS Sustain. Chem. Eng..

[62] Wang L., Xu H., Qiu Y., Liu X., Huang W., Yan N., Qu Z. (2020). Utilization of Ag nanoparticles anchored in covalent organic frameworks for mercury removal from acidic waste water. J. Hazard. Mater..

[63] Afshari M., Dinari M., Zargoosh K., Moradi H. (2020). Novel Triazine-Based Covalent Organic Framework as a Superadsorbent for the Removal of Mercury(II) from Aqueous Solutions. Ind. Eng. Chem. Res..

[64] Xu T., Zhou L., He Y., An S., Peng C., Hu J., Liu H. (2019). Covalent Organic Framework with Triazine and Hydroxyl Bifunctional Groups for Efficient Removal of Lead(II) Ions. Ind. Eng. Chem. Res..

[65] Cao Y., Hu X., Zhu C., Zhou S., Li R., Shi H., Miao S., Vakili M., Wang W., Qi D. (2020). Sulfhydryl functionalized covalent organic framework as an efficient adsorbent for selective Pb (II) removal. Colloids Surfaces A Physicochem. Eng. Asp..

[66] Liang Y., Feng L., Liu X., Zhao Y., Chen Q., Sui Z., Wang N. (2021). Enhanced selective adsorption of NSAIDs by covalent organic frameworks via functional group tuning. Chem. Eng. J..

[67] Zhu X., An S., Liu Y., Hu J., Liu H., Tian C., Dai S., Yang X., Wang H., Abney C.W. (2017). Efficient removal of organic dye pollutants using covalent organic frameworks. AIChE J..

[68] Tang Y., Huang H., Xue W., Chang Y., Li Y., Guo X., Zhong C. (2020). Rigidifying induced fluorescence enhancement in 2D porous covalent triazine framework nanosheets for the simultaneously luminous detection and adsorption removal of antibiotics. Chem. Eng. J..

[69] Park E.Y., Hasan Z., Khan N.A., Jhung S.H. (2013). Adsorptive removal of bisphenol-a from water with a metal-organic framework, a porous chromium-benzenedicarboxylate. J. Nanosci. Nanotechnol..

[70] Hasan Z., Choi E.J., Jhung S.H. (2013). Adsorption of naproxen and clofibric acid over a metal–organic framework MIL-101 functionalized with acidic and basic groups. Chem. Eng. J..

[71] Liu B., Yang F., Zou Y., Peng Y. (2014). Adsorption of phenol and p -nitrophenol from aqueous solutions on metal-organic frameworks: Effect of hydrogen bonding. J. Chem. Eng. Data.

[72] Haque E., Lo V., Minett A.I., Harris A.T., Church T.L. (2014). Dichotomous adsorption behaviour of dyes on an amino-functionalised metal-organic framework, amino-MIL-101(Al). J. Mater. Chem. A.

[73] Zeng T., Yu Y., Li Z., Zuo J., Kuai Z., Jin Y., Wang Y., Wu A., Peng C. (2019). 3D MnO2 nanotubes@reduced graphene oxide hydrogel as reusable adsorbent for the removal of heavy metal ions. Mater. Chem. Phys..

[74] Kireeti K.V.M.K., Chandrakanth G., Kadam M.M., Jha N. (2016). A sodium modified reduced graphene oxide-Fe3O4 nanocomposite for efficient lead(II) adsorption. RSC Adv..

[75] Ezugbe E.O., Rathilal S. (2020). Membrane technologies in wastewater treatment: A review. Membranes.

[76] Slater A.G., Cooper A.I. (2015). Function-led design of new porous materials. Science.

[77] Segura J.L., Mancheño M.J., Zamora F. (2016). Covalent organic frameworks based on Schiff-base chemistry: Synthesis, properties and potential applications. Chem. Soc. Rev..

[78] (2017). Mitch Jacoby 2-D materials go beyond graphene. Chem. Eng. News.

[79] Feriante C.H., Jhulki S., Evans A.M., Dasari R.R., Slicker K., Dichtel W.R., Marder S.R. (2020). Rapid Synthesis of High Surface Area Imine-Linked 2D Covalent Organic Frameworks by Avoiding Pore Collapse During Isolation. Adv. Mater..

